# Long-chain polyunsaturated lipids associated with responsiveness to anti-PD-1 therapy are colocalized with immune infiltrates in the tumor microenvironment

**DOI:** 10.1016/j.jbc.2023.102902

**Published:** 2023-01-13

**Authors:** Mary E. King, Robert Yuan, Jeremy Chen, Komal Pradhan, Isabel Sariol, Shirley Li, Ashish Chakraborty, Oscar Ekpenyong, Jennifer H. Yearley, Janica C. Wong, Luis Zúñiga, Daniela Tomazela, Maribel Beaumont, Jin-Hwan Han, Livia S. Eberlin

**Affiliations:** 1 Department of Chemistry, The University of Texas at Austin, Austin, Texas, USA; 2 Merck Research Laboratories, Merck & Co, Inc, South San Francisco, California, USA; 3 Department of Surgery, Baylor College of Medicine, Houston, Texas, USA

**Keywords:** ambient ionization, desorption electrospray ionization, anti-PD-1, lipid metabolism, immunotherapy, mass spectrometry, molecular imaging, tumor microenvironment, cellular immune response, Cer, ceramide, CL, cardiolipin, DESI, desorption electrospray ionization, FAHFA, fatty acid esters of hydroxy fatty acids, GP, glycerophospholipid, HexCer, hexosylceramide, ICI, immune checkpoint inhibitor, IF, immunofluorescence, LC, liquid chromatography, MALDI, matrix-assisted laser desorption ionization, MS, mass spectrometry, PD-1, programmed cell death protein-1, PE, phosphatidylethanolamine, PG, phosphatidylglycerol, PI, phosphatidylinositol, PS, phosphatidylserine, PUFA, polyunsaturated fatty acid, ROI, region of interest, SAM, significance analysis of microarrays

## Abstract

The programmed cell death protein-1 (PD-1) is highly expressed on the surface of antigen-specific exhausted T cells and, upon interaction with its ligand PD-L1, can result in inhibition of the immune response. Anti-PD-1 treatment has been shown to extend survival and result in durable responses in several cancers, yet only a subset of patients benefit from this therapy. Despite the implication of metabolic alteration following cancer immunotherapy, mechanistic associations between antitumor responses and metabolic changes remain unclear. Here, we used desorption electrospray ionization mass spectrometry imaging to examine the lipid profiles of tumor tissue from three syngeneic murine models with varying treatment sensitivity at the baseline and at three time points post-anti-PD-1 therapy. These imaging experiments revealed specific alterations in the lipid profiles associated with the degree of response to treatment and allowed us to identify a significant increase of long-chain polyunsaturated lipids within responsive tumors following anti-PD-1 therapy. Immunofluorescence imaging of tumor tissues also demonstrated that the altered lipid profile associated with treatment response is localized to dense regions of tumor immune infiltrates. Overall, these results indicate that effective anti-PD-1 therapy modulates lipid metabolism in tumor immune infiltrates, and we thereby propose that further investigation of the related immune-metabolic pathways may be useful for better understanding success and failure of anti-PD-1 therapy.

The development of immune checkpoint inhibitors (ICIs) targeting PD-1 and PD-L1 has transformed the treatment of advanced or metastatic cancers (1, 2). However, only a subset of patients treated with ICIs experience durable cancer remission, with many patients only partially responding or not responding at all. In a growing number of cases, patients become resistant to treatment and develop progressive disease after initially responding (3). Considering the variability of patient response to ICIs, several markers are being explored as a means to predict antitumor response and better inform patient treatment plans. PD-L1 expression, for example, is one of the most widely explored biomarkers for predicting tumor response to ICIs (4, 5, 6). Yet, its effectiveness as a marker is not absolute as different studies have reported that high PD-L1 expression did not reliably predict a treatment benefit for patients, nor did minimal to no expression of PD-L1 preclude a response (7, 8, 9, 10). Another emergent biomarker is tumor mutational burden, which has demonstrated promising results for predicting response independent of PD-L1 (11, 12). Yet, there have also been reports of mutational load failing to correlate with treatment response (13, 14).

It is well established that metabolism plays a major role in modulating the immune system, with mounting clinical evidence indicating that tumor-induced metabolic dysregulation influences treatment outcomes to anti-PD-1 therapy (15, 16, 17, 18, 19). Thus, targeting relevant metabolic pathways may enhance the potency of ICIs in treatments of patients with cancer, a concept that has spurred several ongoing clinical trials (20, 21). As the factors that shape response to ICIs remain unclear, further investigation of how metabolism changes over time following anti-PD-1 therapy may enable identification of new predictive markers and therapeutic targets, as well as offer insight into the design of new combination therapy approaches. Mass spectrometry (MS) is a powerful approach for studying the molecular composition and dysregulated metabolism caused by cancer and other diseases in biological samples. Recently, several MS methods have been used to analyze alterations in metabolites and proteins in serum, plasma, and tissue biopsies from patients following treatment with ICIs (22, 23, 24, 25, 26). For instance, Li *et al.* (22) applied liquid chromatography (LC)-MS to measure changes in metabolite levels in serum from patients with melanoma and renal cell carcinoma before and after treatment with nivolumab and revealed that elevated kynurenine metabolism is associated with resistance to anti-PD-1 and decreased overall survival. Similarly, in a study by Hatae *et al.* (23), LC-MS and gas chromatography coupled to MS were used to study plasma metabolites from patients with non–small cell lung cancer before and after treatment with nivolumab, which allowed identification of four metabolites as potential predictors of response. Harel *et al.* (24) employed LC–tandem mass spectrometry (MS/MS) to analyze protein extracts from tissue biopsies of patients with melanoma treated with immunotherapies including PD-1 blockade, discovering that treatment response was associated with enriched mitochondrial lipid metabolism. While these studies have advanced understanding of the interplay between metabolism and treatment response, little is known about how the lipid profiles of responsive and nonresponsive tumors change in tumor tissue following anti-PD-1 therapy. Probing alterations in lipid species has provided invaluable insight in cancer and immunological research, with recent studies indicating that lipid metabolism shapes antitumor immunity (27, 28). Thus, lipids may be valuable as clinical and therapeutic targets for enhancing the effect of immunotherapies.

MS imaging allows label-free analysis of the spatial distributions of thousands of molecules from a sample, making this approach particularly powerful for visualization and correlation of molecular distributions to histologic structures in tissue (29). Various MS imaging techniques have been used for the molecular and spatial characterization of tumor tissues for numerous applications in cancer research (30, 31). Recently, Berghmans *et al.* (26) applied matrix-assisted laser desorption ionization (MALDI) MS imaging for the analysis of 25 tissue samples from patients with non–small cell lung cancer prior to treatment with ICIs and determined that neutrophil defensins may be biomarkers of treatment response. An alternative MS imaging approach, desorption electrospray ionization (DESI), has become established for the simultaneous detection of hundreds of metabolites and lipid species from tissue, requiring minimal sample preparation and allowing analyses to occur at atmospheric conditions (32). DESI-MS imaging has been extensively used for discovering cancer biomarkers as well as probing dysregulated metabolism in cancerous tissues (33, 34, 35, 36, 37).

In this study, we have applied DESI-MS imaging to characterize the molecular profiles in tumor tissue from syngeneic murine tumor models with the aim of investigating how anti-PD-1 therapy influences and alters metabolism. DESI-MS imaging was used to analyze tissue sections collected at baseline and posttreatment from three tumor models of varying responsiveness to anti-PD-1 therapy. We observed distinct lipid profiles in treated responders compared with control and nonresponders across tumor models and identified hundreds of biomolecules that were either significantly increased or decreased in abundance due to anti-PD-1 therapy. In particular, we discovered that glycerophospholipids containing long-chain polyunsaturated fatty acid (PUFA) chains (referred to here as “PUFA lipids”) were significantly increased in relative abundance over time in anti-PD-1-responsive tumor tissues when compared with baseline and control tumors. Using immunofluorescence (IF) imaging, we found that the signal of PUFA lipids from DESI-MS imaging in the anti-PD-1-responsive tumor largely colocalizes with T cells (CD45^+^ CD3^+^). Overall, our results provide evidence that significant variations in lipid metabolism and specific lipid species are observed in tissues in response to anti-PD-1 treatment, exemplified by an increased PUFA lipid signal in tumor-infiltrating T cells, and thus may be valuable immunometabolic pathways correlating with anti-PD-1 efficacy.

## Results

### DESI-MS imaging of syngeneic models treated with anti-PD-1 therapy

**Figure 1:**
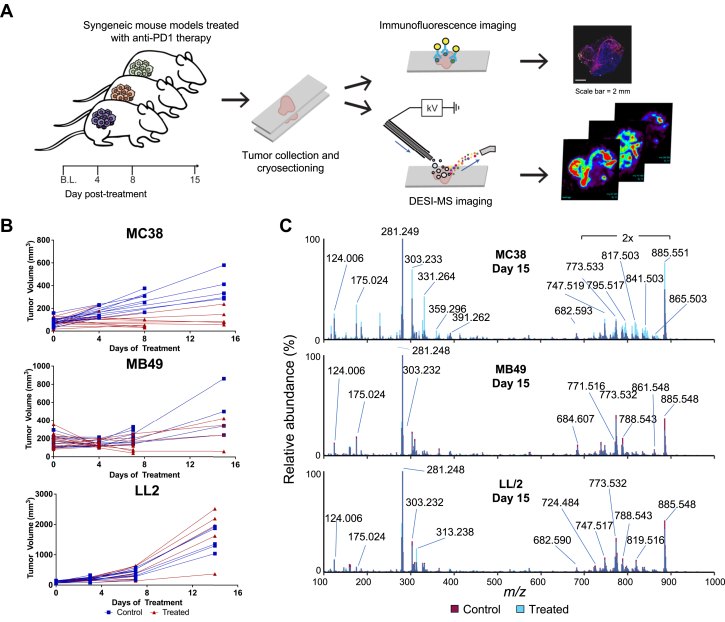
**Desorption electrospray ionization mass spectrometry (DESI-MS) analysis of three syngeneic mouse models of varying treatment sensitivity to anti-PD-1 therapy.***A*, study workflow demonstrating the molecular imaging of tissue from mouse tumor models treated with anti-PD-1 therapy. *B*, tumor growth curves for each syngeneic tumor model. *C*, representative DESI-MS molecular profiles of the lipid mass range obtained from tissues from MC38, MB49, and LL/2 mouse models at posttreatment day 15.

Syngeneic tumor cell lines, MC38 (colon adenocarcinoma), MB49 (urothelial carcinoma), and LL/2 (lung carcinoma), that are highly, moderately, and not responsive to anti-PD-1 therapy, respectively (38), were injected into mice to develop three tumor models for investigating how metabolism is altered in response to anti-PD-1 therapy. Tumor-bearing mice were then treated with anti-PD-1 or isotype control antibodies. Tumors were harvested from mice at the baseline and at three time points post treatment (Fig. 1*A*). Changes in tumor volume at each time point for each model is provided in Figure 1*B*. As shown in Table S1, 92 tumor-bearing mice with n = 3 to 5 in each treatment group were used for this study.

Averaged DESI mass spectra of the lipid mass range (*m/z* 500–1000) obtained from the tissue sections of treated and control groups for each tumor model at final posttreatment day 15 are shown in Figure 1*C*. All averaged mass spectra collected at each time point for both treated and control groups are shown for all three models in Figs. S1–S3. Detection of an assortment of biomolecules, including sphingolipids such as ceramides (Cer), and glycerophospholipids (GPs) including phosphatidylethanolamine (PE), phosphatidylglycerol (PG), phosphatidylinositol (PI), and phosphatidylserine (PS), and cardiolipin (CL) species was achieved. Tentative identifications were made from high mass accuracy measurements and/or tandem MS data.

To explore the molecular differences between treated and isotype control tumors, we first delved into the DESI-MS imaging data collected from the MC38 tumors with therapeutic efficacy shown by reduction of tumor volume following anti-PD-1 treatment. A rich molecular diversity of GP species was detected among both groups, with clear qualitative differences in the molecular profiles of treated responsive MC38 tissues compared with control, most strikingly at posttreatment day 15 (Figs. 1*C* and S2). For example, PG species *m/z* 795.517, *m/z* 817.503, and *m/z* 865.503 were observed in higher relative abundance in the treated responsive tumors than in the control untreated tumor. In control tumors, Cer species such as *m/z* 572.482 and *m/z* 682.593 as well as PG species *m/z* 747.519 and *m/z* 773.533 presented in higher relative abundance compared with treated tumors.

### Statistical analysis of MC38 responsive tissues using significance analysis of microarrays

To more rigorously explore alterations in the molecular profiles due to anti-PD-1 therapy, we next employed the statistical method significance analysis of microarrays (SAM) to identify differences in the relative abundances of detected biological species between the treated and control groups in the MC38 model that were statistically significant. Histopathological evaluation of the same hematoxylin and eosin (H&E)-stained tissue sections analyzed by DESI-MS confirmed the presence and localization of tumor cells as well as regions of stroma and necrosis, enabling us to perform SAM statistical analysis on data extracted from regions of concentrated tumor cells (39). Initially developed to analyze genetic microarray data, SAM has been adapted for use with MS imaging datasets that similarly involve hundreds of data points being collected for one experiment (40, 41, 42). SAM determines statistical significance by calculating a score, *d*, that computes the average change in the normalized peak abundance for each *m/z* between tumor treated with anti-PD-1 therapy and control tumor. As we observed the most obvious differences in the lipid profiles between treated and control at day 15 in MC38, we first performed SAM on this subset of data using a false discovery rate of 0.01.

From the extracted data, SAM selected a total of 103 monoisotopic peaks with biological relevancy with |*d*| ≥ 5, with 69 ions significantly increased in control and 34 ions significantly increased in treated tumor. Positive *d* scores indicate significantly higher relative abundance in treated tumor, whereas negative *d* scores indicate significantly higher relative abundance in control tumors. A comprehensive list of the tentative identifications of SAM features and associated *d* values is provided in Table S2.

**Figure 2:**
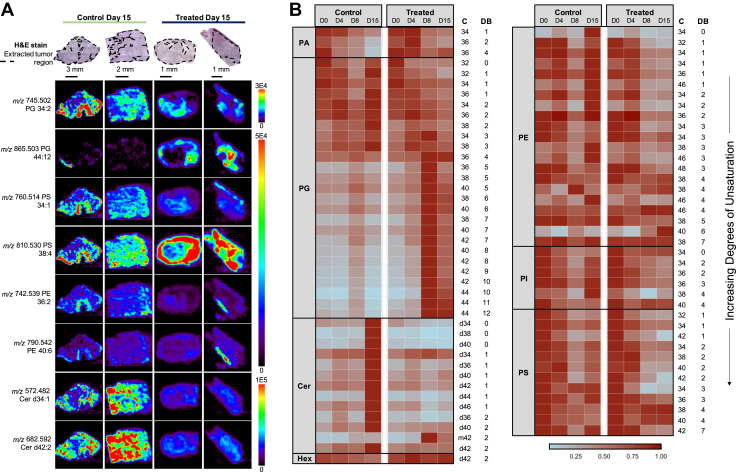
**Alterations in molecular profile of MC38 tumors in response to anti-PD-1 therapy.***A*, desorption electrospray ionization mass spectrometry images of a subset of SAM-selected lipid features from control (*left*) and treated (*right*) tumor samples and corresponding H&E-stained tissue sections. *B*, heatmap illustrating the change in relative abundances of PA, PG, Cer, HexCer, PE, PI, and PS lipid species selected by SAM (|*d*| ≥ 5) over time. SAM was performed on data from mice at posttreatment day 15, but data collected from mice at all time points are plotted. Note that baseline (BL) data are repeated twice to facilitate comparison with molecular changes in both treated and control groups over time. Each row represents the normalized intensities of a unique *m/z* value corresponding to a lipid species, averaged for all samples. C indicates number of carbons and DB indicates number of double bonds, or unsaturations. *Red* indicates highest normalized intensity while *blue* indicates low normalized intensity. All data shown were normalized by the median ion intensity. Cer, ceramide; HexCer, hexosylceramide; PA, phosphatidic acid; PE, phosphatidylethanolamine; PG, phosphatidylglycerol; PI, phosphatidylinositol; PS, phosphatidylserine; SAM, significance analysis of microarrays.

Representative DESI-MS ion images of highly significant SAM features identified as complex lipids detected in tissue at posttreatment day 15 are shown in Figure 2*A*. As reflected in the ion images, SAM revealed remarkable trends in lipid expression related to the degree of polyunsaturation within the fatty acid (PUFA) chains of GP species. From the SAM results, we observed that many highly polyunsaturated GP species were significantly increased in treated tumor across several lipid classes, most notably PGs and also PEs, PIs, and PSs, while more saturated lipid species were significantly increased in control tumor. For example, several long-chain PUFA PG lipids such as PG 40:8 (*m/z* 817.503), PG 42:10 (*m/z* 841.503), and PG 44:12 (*m/z* 865.503) were determined to be significantly increased (|*d*| > 39) in treated tumor compared with control tumor, corroborating trends found in the mass spectra (Fig. 1*C*) and DESI ion images (Fig. 2*A*). Similarly, in control tissue, SAM selected PG species such as PG 32:1 (*m/z* 719.487), PG 34:2 (*m/z* 745.502), and PG 36:1 (*m/z* 775.547) as significantly increased (|*d*| > 18) in relative abundance compared with treated tumor tissues. Other PUFA lipids increased in treated tumor included PS 38:4 (*m/z* 810.530) and PE-NMe2 38:4 (*m/z* 794.572), while more saturated lipids such as PE 36:2 (*m/z* 742.539) and PI 36:2 (*m/z* 861.550) were increased in control tumor. Interestingly, 15 Cer lipids, such as Cer d40:1 (*m/z* 656.576) and Cer d42:2 (*m/z* 682.593), were found to be overall significantly increased (|*d*| > 10) in abundance in control tumor tissue compared with treated tissue irrespective of acyl chain length or degree of unsaturation (Fig. 2*A*).

Following identification of significantly different lipids at posttreatment day 15 between treated and control tumors, we explored how these lipid profiles changed over time from the baseline tumor. To visualize how the abundances of these species shifted with time, we generated a heatmap using the normalized abundances of highly significant lipids averaged for all samples within each treatment group and time point (Fig. 2*B*). Ordering the heatmap by headgroup and increasing degree of unsaturation allowed us to observe a clear shift in PG lipids over time. In addition to being increased in treated tumors at day 15 compared with control tumors, longer-chain PG lipids with 4 to 12 double bonds are in higher abundance at posttreatment day 8 and 15 compared with earlier time points at the baseline and day 4. We then used SAM to evaluate differences in molecular species between treated and control tumors at posttreatment day 4 and day 8, respectively. Overall, 77 molecules were in common between SAM features for treated *versus* control at day 4 and treated *versus* control at day 15, while 71 molecules were in common between SAM results for day 8 and day 15 (Table S2). Interestingly, the longer-chain PUFA PG lipids that were weighted toward day 15 treated tumors compared with control tumors were also determined to be significantly increased at day 4 and day 8 in treated tumors compared with control tumors, respectively.

Alternatively, shorter-chain PG species with 0 to 3 double bonds are in higher relative abundance in control compared with treated. While these species appear to remain in similar relative abundances from baseline to day 15 in control tissues, we observed a continuous decrease in relative abundance of these more saturated PG lipids over time in treated tissues. Similar trends were apparent in PEs, where more saturated lipids with 0 to 3 double bonds were observed to be in higher relative abundance in control tumor at posttreatment day 15. In treated tumor, we observed that more saturated PE species such as PE 34:1 (*m/z* 716.524) generally decreased with time. PS species, on the other hand, generally decreased in relative abundance in tumors post anti-PD-1 therapy compared with baseline tumor, independently of saturation level. The majority of Cer species were relatively increased in control tumor by day 15 compared with baseline and treated tumors.

**Figure 3:**
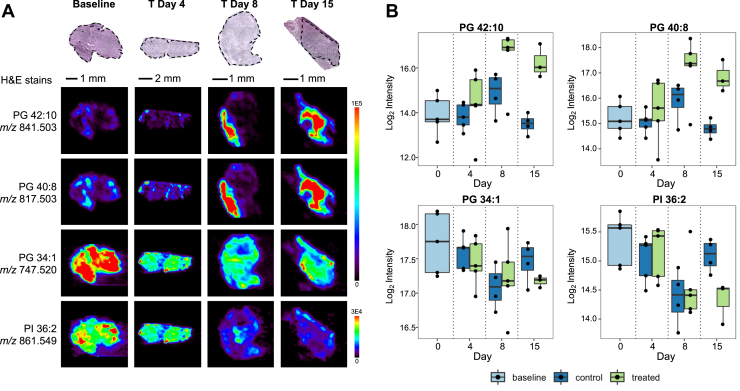
**Molecular alterations between baseline, treated, and control MC38 tumors over time due to anti-PD-1 therapy.***A*, representative desorption electrospray ionization mass spectrometry ion images of MC38 tumors at baseline and each posttreatment time point of several lipids that are highly significantly different between baseline and treated tumor at posttreatment day 15, as well as between treated and control groups at each of the three time points post treatment. Note that the same sample (H&E stain and associated desorption electrospray ionization ion images) representing treated tumors at posttreatment day 15 group shown here is also shown in Figure 2*A* to represent the same group. *B*, box plot of the log_2_ transformed intensities of the lipids shown in *A* for baseline, treated, and control tumor groups.

We then used SAM to determine if there were statistically significant differences in lipids between baseline tumor tissue and posttreatment day 15. A total of 83 identified molecules with |*d*| ≥ 5 were determined as significantly different between these groups, where positive *d* scores indicate significantly higher relative abundance in day 15 treated tumor and negative *d* scores indicate significantly higher relative abundance in baseline tumors. Tentative identifications of significant ions are provided in Table S3. For baseline tissues, 49 ions were significantly increased compared with treated tissue, while 34 ions were significantly increased in treated tissues in contrast to baseline tissue. Notably, 65 molecules (78% of the significant molecules) were in common between SAM results for baseline *versus* treated tumor at day 15 and treated *versus* control tumor at day 15. Figure 3*A* shows DESI-MS images of various features that were significant for these groups as well as posttreatment day 4 and day 8 treated *versus* control tumors plotted in representative tumors tissues at each time point. For example, PG 42:10 (*m/z* 841.503) and PG 40:8 (*m/z* 817.503) were significantly increased in treated tumors compared with control tumors at posttreatment days 4, 8, and 15 as well as baseline tumors compared with treated tumors at day 15. As shown in Figure 3*B*, boxplots of the log_2_ normalized intensities revealed that both lipids increase over time in treated tissues and are highest on posttreatment day 8. In contrast, baseline tumors had significantly higher relative abundances (|*d*| > 20) of lipids such as PG 34:1 (*m/z* 747.519) and PI 36:2 (*m/z* 861.550) compared with treated tumors. Comparing treated with control tumors within each time point, PG 34:1 was determined to be weighted toward control tumors at day 4 (*d* = −9.58) and treated tumors at day 8 (*d* = 4.72), while PI 36:2 was weighted toward treated tumors for both day 4 (*d* = 16.40) and day 8 (*d* = 2.47). Interestingly, both lipids were significantly increased in control tumors at posttreatment day 15 compared with treated tissues.

To examine how anti-PD-1 treatment may influence lipid metabolism in other major immune organs, tissue sections from spleen and tumor draining lymph nodes obtained from the same mice were also imaged using DESI-MS. For spleens at posttreatment day 15, SAM selected 69 biological species in common with features identified as significantly different between MC38 treated and control tumors. Fig. S5 shows representative DESI-MS ion images of PG species that are significantly different between treated and control spleens. Compared with the tumors, the spleens do not exhibit as clear qualitative differences in PUFA lipid signal between treated and control groups based on the DESI-MS images. Furthermore, the SAM *d* scores are generally less significant for spleens compared with the *d* scores obtained for the tumors (Table S2). For example, PG 44:12 (*m/z* 865.503) is significantly increased in treated spleens compared with control spleens with a *d* score of 5.12, while the same lipid is significantly increased in treated tumors compared with control tumors with a greater *d* score of 39.94. Similarly, SAM was performed on data from posttreatment day 15 tumor-draining lymph nodes to assess molecular differences in treated and control tissues. Overall, 73 species were found in common with results obtained for comparing MC38 tumors at the same time point, with representative DESI-MS ion images of a subset of these SAM features shown in Fig. S6. Interestingly, trends regarding relative abundance of PUFA lipids in the tumor-draining lymph nodes are similar to those observed in the tumors, particularly among PG species. For example, PG 44:11 (*m/z* 867.516) and PG 40:6 (*m/z* 821.531) are significantly increased in treated lymph nodes (*d* = 16.25, 15.64) and treated tumors (*d* = 38.64, 26.80), respectively, compared with the control tissues, highlighting tumor-associated metabolic changes consistently found in immune compartments of both tumors and their draining lymph nodes.

### Multiplex immunofluorescence imaging of MC38 tumor tissues

**Figure 4:**
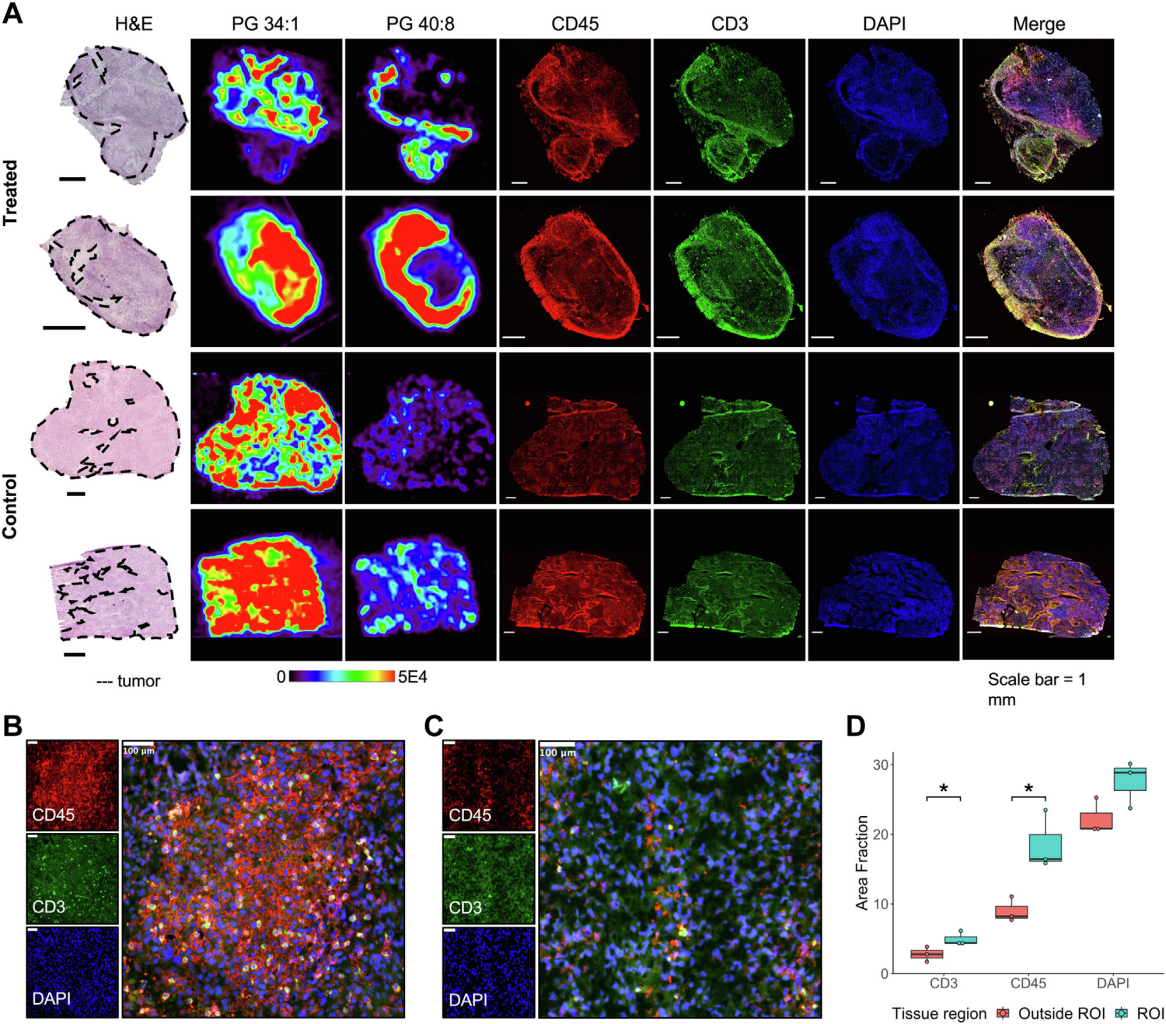
**Immune cells spatially colocalize with polyunsaturated lipid species in MC38 tumors.***A*, comparison of desorption electrospray ionization mass spectrometry ion and immunofluorescence images from MC38 tumors. Markers for CD3 are shown in *green*, CD45 in *red*, and DAPI in *blue*. Brightness and contrast were uniformly enhanced across each channel for all immunofluorescence images in Fiji for improved visualization. A high-magnification view of representative (*B*) control tumor and (*C*) treated tumor within the region of interest (ROI) based on high desorption electrospray ionization mass spectrometry signal of polyunsaturated fatty acid lipids. *D*, the area fraction of immune cell and nuclear markers within the ROI selected based on high polyunsaturated fatty acid lipid signal and outside the ROI in treated tumor tissue (∗*p* < 0.05).

We observed that the DESI-MS images of PUFA lipids in MC38 treated tumors were highly heterogeneous, with no clear localization to demarcated regions of tumor within the same H&E-stained tissue section (Figs. 2*A* and 3*A*). To further characterize the cellular landscape of these tumors, we performed multiplex IF staining on all frozen serial tissue sections (n = 7) from MC38 treated and control tumors at posttreatment day 15. We used CD45 as a general marker for immune cells and CD3 as a marker for pan-T cells, with DAPI as the nuclear counterstain. Representative IF images of whole tissue sections compared with DESI-MS images and optical images of H&E stain obtained from serial tissue sections are shown in Figure 4*A*. Notably, we observed that the unique spatial distribution of PUFA lipids as detected in the DESI-MS ion images spatially colocalize with CD3 and CD45 immune cell markers in the IF images. To illustrate this colocalization, we created a short animation of a DESI-MS image of PG 40:8 overlaid onto the merged IF image of a serial tissue section (Movie S1). In contrast, the spatial distributions of more saturated lipids, such as PG 34:1 illustrated in Figure 4*A*, do not appear to closely overlap with immune cells. Higher magnification of the IF images for representative control and treated tumor tissue is provided in Figure 4, *B* and *C*, respectively, demonstrating the increased density of CD3 and CD45 cells in treated compared with control tumor.

To quantify and compare the level of immune cell infiltration in regions of both high and low DESI-MS signal of PUFA lipids in treated tissues, we calculated the relative area of pixels above a uniform intensity threshold for CD3, CD45, and DAPI markers. Regions of interest (ROIs) were delineated based on high PUFA lipid signal in the DESI images. Figure 4*D* shows a box plot comparing area fraction of each marker within the ROI and outside the ROI in treated MC38 tumors. CD3 and CD45 markers were statistically significantly increased (*t* test, *p*-value < 0.05) within the ROI compared with outside the ROI. These results corroborate our visual findings, indicating regions of high immune cell infiltration overlap with regions of high DESI-MS signal from PUFA lipids in treated MC38 tumors.

### Analysis of MB49 and LL/2 models of anti-PD-1 therapy

**Figure 5:**
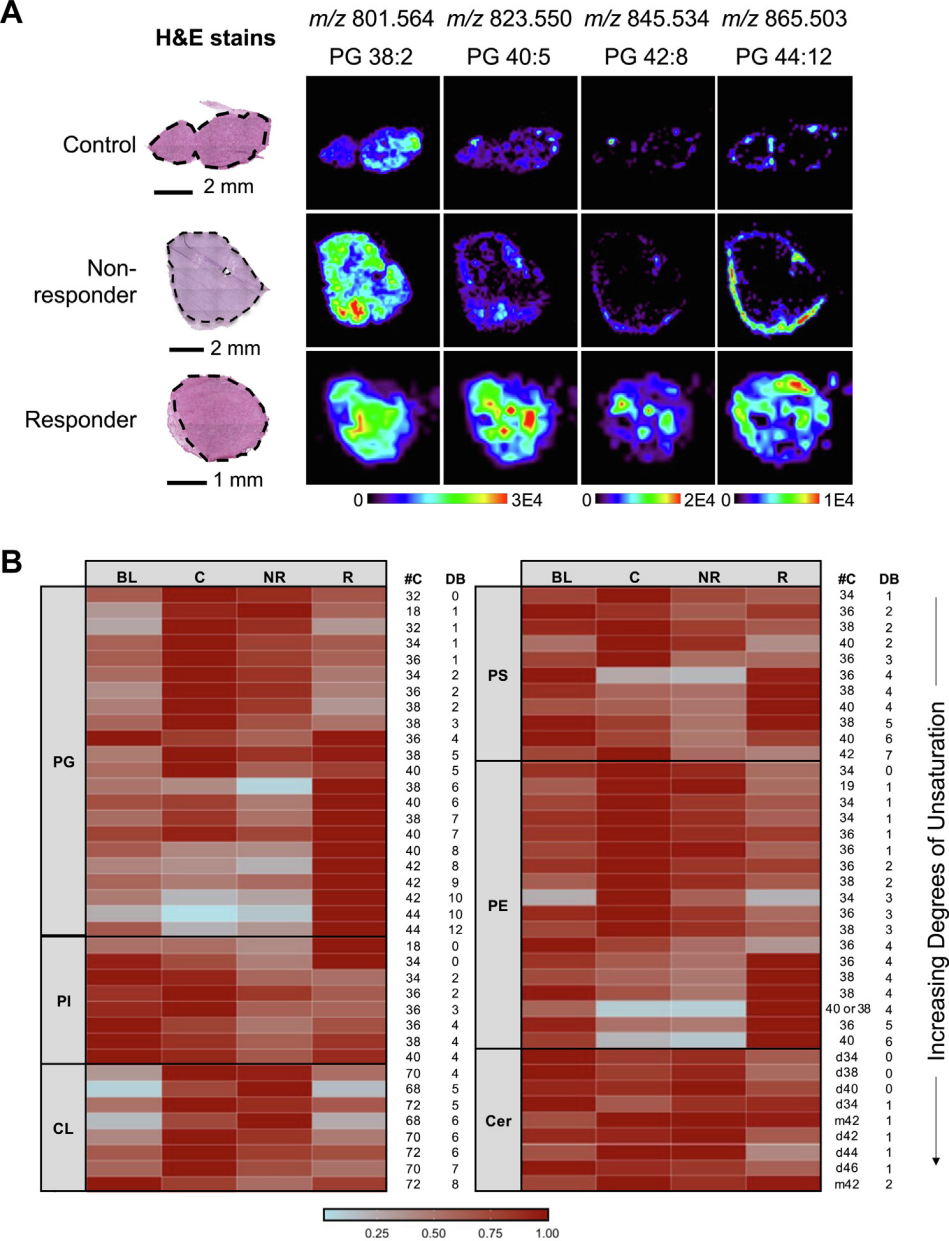
**Comparison of lipid profiles of treatment-responsive, treatment-nonresponsive, and control tumors in the MB49 anti-PD-1 treatment model.***A*, desorption electrospray ionization mass spectrometry images of a subset of SAM-selected lipid features from responsive (R) treated tumor, nonresponsive (NR) treated tumor, and control (C) tumor at posttreatment day 15 and corresponding H&E-stained tissue sections. *B*, heatmap illustrating the change in relative abundances of CL, PE, PG, PI, PS, and Cer lipid species selected by SAM (|*d*| ≥ 5). SAM was performed on data from all responders and nonresponders regardless of time point, but data collected from mice at the baseline and from control groups are plotted as well for comparisons. Each row represents the normalized intensities of a unique *m/z* value corresponding to a lipid species, averaged for all samples. #C indicates number of carbons and DB indicates number of double bonds, or unsaturations. Cer, ceramide; CL, cardiolipin; PE, phosphatidylethanolamine; PG, phosphatidylglycerol; PI, phosphatidylinositol; PS, phosphatidylserine; SAM, significance analysis of microarrays.

We next evaluated the DESI-MS imaging data collected from two additional mouse tumor models, MB49 (mildly responsive to anti-PD-1) and LL/2 (nonresponsive to anti-PD-1). Within the treated group of MB49 urothelial carcinoma tumors, there were responders as well as nonresponders at each time point following treatment. Three of four tumors responded to treatment at day 4, two of three tumors responded at day 8, and one tumor of four responded at day 15 based on tumor growth data (Fig. 1*B*). Figure 5*A* shows representative DESI-MS ion images of MB49 tumors comparing a control, nonresponder, and responder tumor at posttreatment day 15. Remarkably, PUFA lipids such as PG 42:8 (*m/z* 845.534) and PG 44:12 (*m/z* 865.550) were present in increased relative abundance in the responder compared with nonresponders and control tumors at posttreatment day 15, similar to what was observed from DESI-MS images and statistical results in MC38 treatment responsive tumors.

As the limited sample size for treatment outcome precluded us from performing statistical analysis to identify molecular differences between responders and nonresponders at each time point, we performed SAM on all tumor responders (N = 6) *versus* nonresponders (N = 5) irrespective of time. Overall, 90 lipids were identified as significantly different between responding and nonresponding tumors with |*d*| ≥ 5, with 72 species in common with SAM results for MC38 treated *versus* control tumors at posttreatment day 15 (Table S4). Similar to the results observed between treated and control tumors in the MC38 model, several longer-chain PUFA lipids were significantly increased across multiple lipid classes in treated responders compared with nonresponders within the MB49 tumor model regardless of posttreatment time point. A subset of these lipids, including CL, Cer, HexCer (hexosylceramide), PE, PG, PI, and PS species, are plotted in a heatmap (Fig. 5*B*) comparing all baseline, control, nonresponder, and responder tumors and organized by increasing degree of unsaturation similar to Figure 2*B*. Among these lipids, the relative abundance of PG, PE, and PS species with four or more total unsaturations appear to be generally increased in responders compared with nonresponders as well as control and baseline tumors. CL species, the majority of which are significantly increased in nonresponders compared with responders, appear to be generally increased in control tumors compared with baseline tumors as well.

Finally, we evaluated if there were molecular differences present in the nonresponsive LL/2 lung carcinoma model to gain insight into alterations in metabolism that occur due to treatment rather than tumor response. We applied SAM to LL/2 DESI-MS data from treated and control groups at posttreatment day 15 and tentatively identified 98 ions as monoisotopic peaks that were selected as significantly different between these groups. Of these, 28 were significantly increased in nonresponsive treated tumors and 70 were significantly increased in control tumors. Interestingly, 11 cardiolipin species such as CL 72:6 (*m/z* 725.491) and CL 70:5 (*m/z* 712.483) were highly significantly increased (*|d|* > 45) in control tumors compared with nonresponding treated tumors as shown in Fig. S7*A*. A heatmap plotting the normalized intensities of all SAM-selected CL lipids for each time point from baseline to posttreatment day 15 in Fig. S7*B* reveals that all CLs increased in relative abundance over time in control tumors compared with treated tumors. In comparison, treated tissues had five significantly increased (|*d*| > 39) fatty acid esters of hydroxy fatty acid (FAHFA) species such as FAHFA 34:1 (*m/z* 535.472) and FAHFA 36:1 (*m/z* 563.503) compared with control tissues. Although some long-chain PUFA PG lipids were identified by SAM as significantly different between treated and control groups, these lipids were weighted toward both groups, in contrast to MC38 and MB49 models were PUFA lipids were increased near exclusively in treated responders.

## Discussion

Altered lipid metabolism in the tumor microenvironment has been shown to influence patient outcomes to treatment with PD-1 inhibitors (28, 43, 44). As such, lipids are posited as potential therapeutic targets to boost immune response to treatment. Here, we have applied DESI-MS imaging with SAM to study alterations in lipid species following anti-PD-1 therapy in three syngeneic mouse models of varying treatment sensitivity at the baseline and three posttreatment time points. We discovered that long-chain PUFA lipids, particularly PG species, tend to increase in relative abundance over time compared with baseline and control tumors of MC38 colon cancer model following anti-PD-1 therapy. Notably, we observed similar lipid profiles in treatment responders compared with nonresponders and control tumors in the MB49 urothelial cancer model, suggestive of a broad trend in immunometabolic changes regardless of the type of primary tumors, driven by anti-PD-1 therapy. DESI-MS imaging together with IF imaging confirmed that the spatial distribution of these PUFA lipid species colocalize with dense regions of infiltrating immune cells in treated responsive MC38 tumors.

SAM enabled selection of 577 ions as significantly different in the lipid mass range between anti-PD-1 treated and control MC38 colon cancer tissues at posttreatment day 15. Of these ions, we identified 103 tissue-specific biological species (Table S2). For subsequent statistical comparisons between baseline (day 0) and treated (day 15) tissues, SAM selected a total of 480 ions of which 83 were similarly identified as biologically relevant species. Notably, from these molecules we identified a total of 65 significant features in common with SAM features selected for characterization of treated and control MC38 tissues. Aside from the features tentatively identified using high mass accuracy (≤5 ppm) and/or tandem MS measurements, the remaining number of features were attributed to non-tissue-specific ions, isotopes of already selected molecular ions, and/or peaks that were not matched based on accurate mass within METASPACE annotation platform for MS imaging data (45). Note that characterization of the double bond location within the fatty acid chains of lipids using collision-induced dissociation MS was not possible, preventing precise structural assignment of lipid isomers.

Among the SAM features, multiple PG species were selected as highly significant for characterizing MC38 treatment responders compared with control tumors. Zare *et al.* also identified alterations in PGs between normal and cancerous tissue in MYC-driven renal cell carcinoma and lymphomas in transgenic mouse models using DESI-MS imaging (41, 46). Although a minor component (∼1%) of animal cell membranes, PGs predominantly serve as precursors for CL biosynthesis, which are localized to mitochondrial membranes (47). In our study, PGs with more saturated fatty acid chains were characteristic of control and baseline tumors, whereas more polyunsaturated PGs were indicative of MC38 tumor responders at each time point post treatment (Figs. 2 and 3). The degree of lipid saturation profoundly affects immune system functions as well as membrane fluidity. It has been well established that memory T cells rely on mitochondria-derived fatty acid metabolic pathways, while effector T cells use both glycolysis and oxidative phosphorylation (48). We speculate that the relatively increased PUFA lipid signal in intratumoral T cells within anti-PD-1-responsive MC38 are indicative of a metabolic transition toward memory-like T cells in the process of tumor antigen clearance. Of note, recent studies provide evidence to a link between PUFAs and the microbiome in enhancing the immune system’s response to ICIs (49, 50). Furthermore, as a higher degree of unsaturated FAs causes cell membranes to be more prone to lipid peroxidation and subsequent cell death, cancer cells generally have a lower degree of unsaturation (51, 52). In addition, ceramides were generally found to be significantly increased in control tumors compared with treated tumors at the final time point post treatment (day 15). Interestingly, ceramides play important and opposing roles in tumor progression and suppression, which are largely dependent on acyl chain length and subcellular localization (53).

With DESI-MS and IF imaging of serial tissue sections, we discovered a unique PUFA lipid signature associated with MC38 treatment response that spatially overlaps with tumor immune infiltrates (Fig. 4). Based on the H&E-stained tissues compared with IF images, some regions of T cells overlap with tumor cells in treated tissue sections. Note that the spatial resolution employed for DESI-MS imaging experiments (150 μm) does not allow for precise attribution of the molecular profiles to tumor cells or immune cells within these mixed histological regions. However, the relative area of CD3- and CD45-stained immune cells within the region of colocalizing PUFA lipid signal is significantly higher compared with the rest of the tissue (Fig. 4*D*), providing evidence that this lipid signature is related to lipid composition in plasma membranes of immune infiltrates, such as T cells, as opposed to tumor cells. There are potentially other immune infiltrates in addition to T cells that may also be related to these lipid alterations, given the increased positive staining of CD45 compared with CD3. To determine the specific immune cell populations that contribute to these PUFA lipid alterations, we performed experiments to evaluate the lipid profiles of CD4 and CD8 cells isolated from MC38 tumors (see Supporting Information). However, these experiments were inconclusive due to low mass spectra signal obtained and will be explored further (Fig. S8). In addition, analysis of the relationship between the quantity of immune cell subtypes and lipid changes while considering the spatial distribution of cells within the tumor microenvironment should also be performed in future studies. In addition, DESI-MS imaging and statistical analysis of draining lymph nodes proximal to tumors indicated that several PUFA PG lipids were also significantly increased in treated lymph nodes compared with controls. Interestingly, a preclinical study by Fransen *et al.* (54) reports that tumor-draining lymph nodes contribute to the activation and regulation of antitumor immune responses and is associated with improved response to anti-PD-1 therapy. As the functional role of these PUFA lipids in immune processes remains unclear, further experiments will be performed to explore the translational potential of these molecules as future treatment biomarkers and therapeutic targets in combination with anti-PD-1 therapy. A limitation of our study is the lack of clinical data from human samples to validate our preclinical findings, which will be pursued in future studies. Mechanistic studies will also be investigated to further investigate the nature of the association between these lipid alterations and treatment response.

We also observed that the increase in relative abundance of PUFA lipids appears to extend to anti-PD-1 responders within the MB49 urothelial cancer model based on SAM results comparing all responding tumors to nonresponding tumors and, as illustrated in Figure 5, although larger sample sizes are needed for proper statistical evaluation at each time point. Similar to our observations, Mock *et al.* (25) discovered that long-chain PUFA lipids detected in serum were predictive of response to ICIs in 28 human patients with renal cell carcinoma or bladder urothelial carcinoma. However, while our DESI-MS imaging results show a relative increase in PUFA lipids at posttreatment day 15 associated with response compared with no response, Mock *et al.* found that baseline levels of lipids were the most important for successful prediction of treatment outcome. Given that each mouse was sacrificed following tumor resection at each time point in our study, the final treatment outcome for MB49 baseline tumors is unknown, preventing proper evaluation of baseline molecular data. Interestingly, for our LL/2 lung cancer model of no treatment response, we observed trends among CL species in the DESI-MS images and SAM features between control and treated tumors (Fig. S7). CLs were present in significantly lower relative abundances in control tumor compared with treated tumor. As vital components of mitochondrial membranes in animal tissues, CL species have been widely implicated in various human diseases including cancer and, more recently, modulating immune system processes and cell death (55, 56). Determining if these differences in CL profiles between control and treated tissue are due to altered CL content within the mitochondria or if there is a greater number of mitochondria within control tumor tissue remains to be explored. Offering a valuable opportunity to uncover mechanisms of resistance as well as therapeutic targets, additional molecular characterization of nonresponsive tumors will also be pursued. Furthermore, how other antitumor therapeutic approaches, such as chemotherapy, radiotherapy, targeted therapy, or additional immunotherapy beyond anti-PD-1, can metabolically potentiate anti-PD-1 therapy in a combination approach warrants boarder and deeper future research.

Our results demonstrate that DESI-MS imaging is valuable for studying molecular alterations and immune responses in the tumor microenvironment of biological tissue over time due to treatment with ICIs. A longitudinal study comparing baseline to posttreatment levels within the same subject as well as DESI-MS analysis of human tissue biopsies is necessary to further explore and validate the findings described in this study. In addition, investigation of the functional roles of these molecules will be pursued in subsequent studies. Overall, these lipid markers may provide useful information on treatment resistance as well as lead to discovery of new therapeutic targets for improving response to anti-PD-1 therapy.

## Experimental procedures

### Murine samples

MC38, MB49, and LL/2 syngeneic cell lines were passaged two times after being thawed until implantation. One million viable cells of each cell line were implanted in the right flank of wildtype C57BL/6 mice (Jackson Laboratory). Mice with tumor formation were enrolled in the study when the average tumor volume reached 100 mm^3^. MRL’s anti-murine PD-1 antibody (clone DX400, mouse IgG1-[D265A]) and its isotype control antibody (clone TC31-27F11.C2; specific for hexon protein, human adenovirus 5) were injected intraperitoneally at 10 mg per kg dose every 4 days as indicated previously (57). All animal procedures were approved by the IACUC of Merck & Co, Inc, in accordance with Association for Assessment and Accreditation of Laboratory Animal Care (AAALAC) guidelines.

### Tumor sample preparation for DESI-MS and immunofluorescence experiments

Tumor (n = 95), spleen (n = 95), and draining lymph node (n = 90) samples from each tumor model were prospectively collected from MRL at the baseline and from mice treated with anti-PD-1 or isotope control at days 4, 8, and 15 post treatment (Table S1). Tissue samples were sectioned at 5- and 8-μm thickness for DESI-MS and IF experiments, respectively, using a CryoStar NX50 (Thermo Scientific). Tissue sections were mounted on a glass slide and stored in a −80 °C freezer until analysis. Sections from three treated samples, MC38 T1 and T5 at posttreatment day 15 and MB49 T4 at posttreatment day 8, had a complete absence of tumor cells as determined by pathological evaluation and thus were excluded from statistical analysis and subsequent experiments, resulting in a final tumor sample size of n = 92.

### DESI-MS imaging

All DESI-MS analyses were performed using a laboratory-built DESI sprayer fitted to a Thermo Orbitrap Q-Exactive HF mass spectrometer at a resolving power of 60,000 in the negative ion mode in the mass range *m/z* 100 to 1500. Tissue sections were thawed and dried in a chemical fume hood 10 min prior to DESI-MS analysis. DESI-MS imaging of tissue sections was performed at a spatial resolution of 150 μm using a spray flow rate of 1.5 μl min^−1^ with a histologically compatible solvent system, acetonitrile:dimethylformamide 3:1 (v/v). The pressure of the nebulizing N_2_ gas was set to 180 psi. DESI-MS ion images were constructed using FireFly (Thermo Scientific) and BioMAP (Novartis) software. Ion identifications were made with high mass accuracy measurements (≤5 ppm mass error) and/or tandem MS experiments (Figs. S4 and S9) using high-energy collision dissociation. METASPACE and LIPID MAPS databases were used to aid in molecular identification.

### Pathological evaluation

The same tissue sections analyzed with DESI-MS were stained using standard H&E protocol. Pathological evaluation was performed by J. H. Y. using light microscopy, where regions of concentrated tumor cells, necrosis, and/or fibrosis were annotated on H&E-stained sections. Optical images of H&E-stained tissues were taken using an Invitrogen EVOS FL Auto 2 Imaging System (Thermo Scientific) in brightfield.

### Immunofluorescence analysis

To determine the spatial distribution of immune cells in tissue sections adjacent to those analyzed by DESI-MS, frozen tumors were sliced at 8 μm and fixed in 2% paraformaldehyde for 10 min. Tissue sections were then washed in PBS and blocked with 5% goat serum (Dako) in 1% bovine serum albumin. After blocking, the sections were incubated overnight at 4 °C with rat anti-CD45 (Biolegend, clone 30-F11) and rabbit anti-CD3 (Abcam, ab16669) diluted 1:100 in 5% goat serum in 1% bovine serum albumin. After washing, sections were incubated with goat anti-rat Alexa Fluor 488 (Thermo Fisher) and goat anti-rabbit Alexa Fluor 594 (Thermo Fisher) for 1 h at room temperature. Slides were then washed and mounted with Permafluor (Thermo Fisher) and imaged using a Keyence BZ-X810 widefield fluorescent microscope. Images were analyzed using ImageJ.

All processing and quantitation of fluorescence in microscopy images of tissues were performed using Fiji image processing software (58). Brightness and contrast were uniformly adjusted for enhanced visualization of IF images. To compare area fraction of positively stained cells within regions of high PUFA signal to low PUFA signal in treated tissues, the Otsu thresholding algorithm was applied to raw images after background subtraction (59).

### Statistical analysis

Pixels, or mass spectra, corresponding to regions of concentrated tumor cells within tumors, white pulp in spleen tissue, or lymph node tissue were extracted using MSiReader software (60), excluding any pixels with fibrosis and necrosis. Extracted data were imported to RStudio for remaining preprocessing steps and subsequent statistical analysis. Prior to SAM analysis, the mass range of mass spectra was restricted to *m/z* 500 to 1000. Mass spectra were then binned to 0.01 *m/z*, background was subtracted, and peaks appearing in fewer than 10% of pixels were removed. The data were then normalized to the median nonzero ion intensity. SAM was employed using the “samr” package in the CRAN R library to identify molecules with significantly different relative abundances between groups within each tumor model with a false discovery rate of 0.01 (42).

Following quantitation of mean intensity and area fraction of IF data, significance testing *via t* tests was performed in RStudio to determine if differences between groups were significant (*p*-value < 0.05).

## Data availability

MS data for this study have been deposited on Harvard Dataverse and can be found at https://doi.org/10.7910/DVN/Z3QMLC.

## Supporting information

This article contains supporting information.

## Conflict of interest

L. S. E. is an inventor in patents owned by Purdue Research Foundation related to DESI-MS imaging technology and receives royalties from the commercialization of the technology. R. Y., J. C., O. E., J. H. Y., J. C. W., L. Z., D. T., M. B., and J.-H. H. are employees of Merck Sharp & Dohme Corp, a subsidiary of Merck & Co, Inc, Kenilworth, NJ, USA, and shareholders in Merck & Co, Inc, Kenilworth, NJ, USA.
