# Supplementary material for: Long-chain polyunsaturated lipids associated with responsiveness to anti-PD-1 therapy are colocalized with immune infiltrates in the tumor microenvironment
Source: J Biol Chem. 2023 Jan 13;299(3):102902. doi: 10.1016/j.jbc.2023.102902 (PMC9957763; doi:10.1016/j.jbc.2023.102902)
Supplement: Supplemental Figures S1–S9 and Tables S1–S4 [file mmc1.docx]

**Supporting Information**

**Long-chain polyunsaturated lipids associated with responsiveness to anti-PD-1 therapy are co-localized with immune infiltrates in the tumor microenvironment**

**Authors:** Mary E. King^1^, Robert Yuan^2^, Jeremy Chen^2^, Komal Pradhan^2^, Isabel Sariol^1^, Shirley Li^1^, Ashish Chakraborty^1^, Oscar Ekpenyong^2^, Jennifer H. Yearley^2^, Janica C. Wong^2^, Luis Zúñiga^2^, Daniela Tomazela^2^, Maribel Beaumont^2*^, Jin-Hwan Han^2*^, and Livia S. Eberlin^1,3*^

**Affiliations:**

^1^ Department of Chemistry, The University of Texas at Austin, Austin, TX 78712.

^2^ Merck Research Laboratories, Merck & Co., Inc., South San Francisco, CA 94080, USA.

^3^ Department of Surgery, Baylor College of Medicine, Houston, TX 77030

*Co-corresponding authors: MB: [maribel.beaumont@merck.com](mailto:maribel.beaumont@merck.com) ; JHH: [jin-hwan.han@merck.com](mailto:jin-hwan.han@merck.com) ; LSE: [livia.eberlin@bcm.edu](mailto:livia.eberlin@bcm.edu)

**Supplemental results.**

To further determine the specific immune cell populations that contribute to these lipid alterations, we conducted several rounds of experiments with MC38 tumor models. First, MC38 mouse models treated with anti-PD-1 therapy (n=2) were generated. Cells were isolated from the tumors and sorted using fluorescence-activated cell sorting into CD4 and CD8 immune cells. The sorted populations were pelleted, frozen into OCT blocks, sectioned at 12 µm thickness, and analyzed using DESI-MS for assessment of lipid profiles. While some lipid species were detected from the sections, the signal intensities were low and only the most abundant lipid species commonly detected with DESI-MS, such as PI 38:4 at *m/z* 885.549 were detected with S/N>3, as shown **Figure S8**. For reference, in tissue samples, polyunsaturated fatty acid lipids of interest such as PG 44:12 at *m/z* 865.501 and PG 44:11 at *m/z* 867.518 were detected in lower relative abundance compared to other lipids (**Figure S8**). While PG 44:11, a PUFA lipid significantly increased in relative abundance in treated responsive MC38 tumors compared to controls, was tentatively observed in CD4 cells for one sample, the S/N was below 3, thus below detectable levels. We attribute the lack of detection of PUFA lipids of interest to the low number of cells present in the cell pellets (16,000-43,000). We repeated the experiment with n=5 mice (resulting in est. cell count 1,000-25,000 per pellet), but similarly only detected a few lipids in a subset of the samples.

Lipid extracts from leftover cell pellets were generated and the resulting extracts were analyzed using direct ESI-MS infusion. However, interference due to residual OCT in the sample caused signal suppression and hindered analysis. We repeated the experiment again with additional mice (n=5) and generated fresh frozen cell pellets from the isolated cell populations (est. count 200-44000). We created lipid extracts from these samples and analyzed them using ESI-MS but observed low biological signal intensities again, with only a few of the most abundant lipids detected in tissue also observed in the mass spectra for the pellets. Future experiments in a follow-up study will be pursued to gain further insight into the types of immune cells corresponding to these lipid alterations.

**Fig. S1.** Averaged mass spectra of MC38 anti-PD-1 model at the baseline and three time-points post-treatment.

**Fig. S2.** Averaged mass spectra of MB49 anti-PD-1 model at the baseline and three time-points post-treatment.

**Fig. S3.** Averaged mass spectra of LL/2 anti-PD-1 model at the baseline and three time-points post-treatment.

**
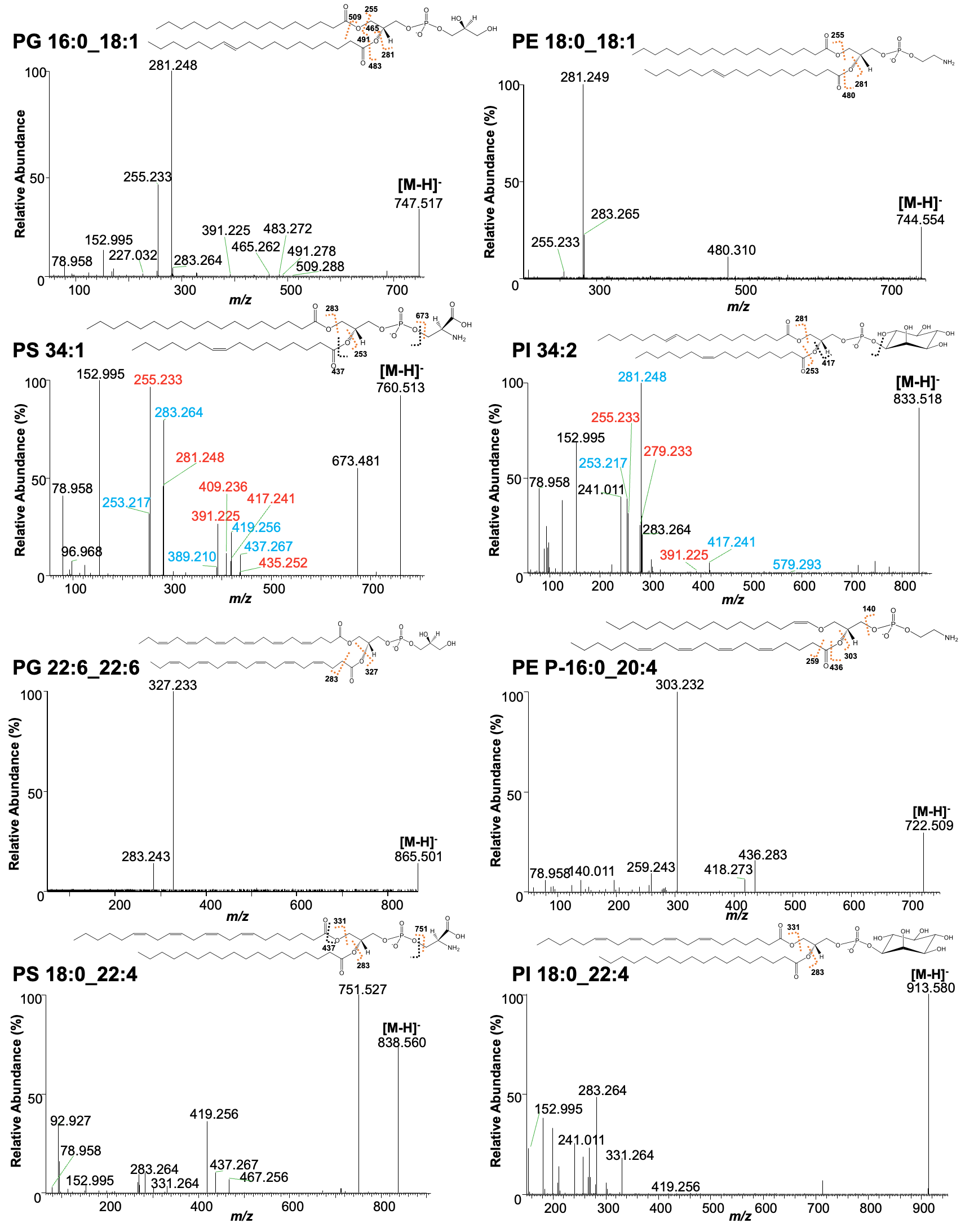
**

**Fig. S4.** Example tandem mass spectra of glycerophospholipids detected in MC38 model which were significantly different between control and treated tumor both at day 15. Note that fragments corresponding to isomeric lipids with different fatty acid compositions are denoted in red or blue. For isomers, the structure shown only corresponds to the fragments denoted in blue.


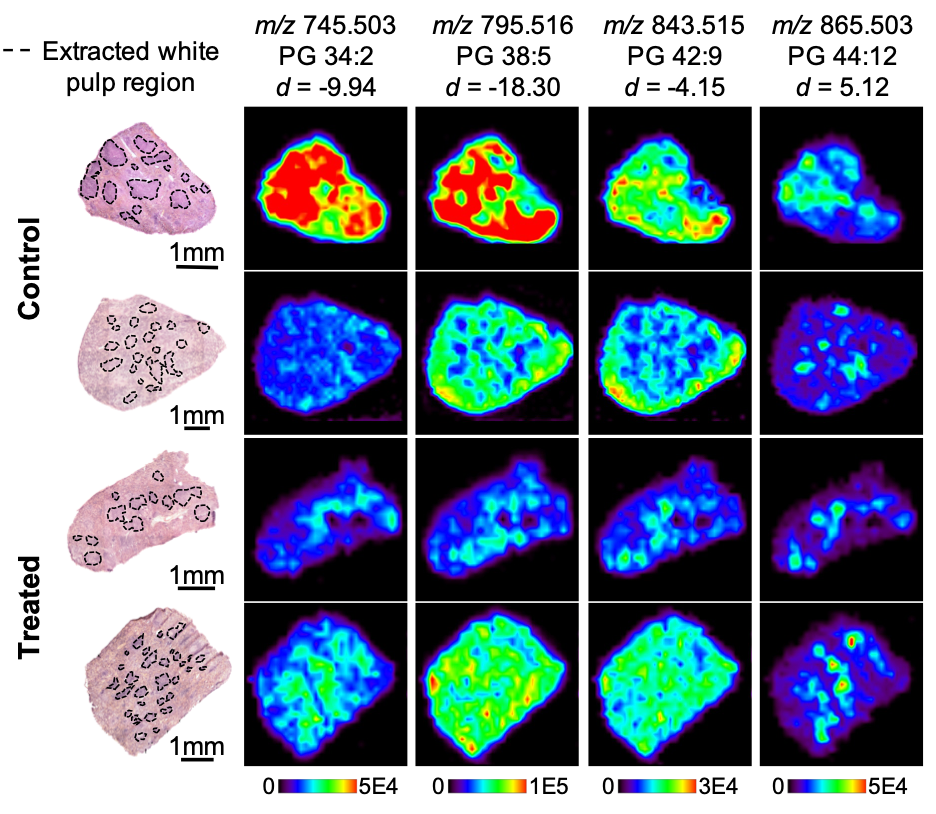


**Fig. S5.** Representative DESI-MS ion images of a subset of PG species that were selected by SAM as significantly different between MC38 treated and control spleen tissues at post-treatment day 15 and also in common with tumor tissues from mice within the same group.


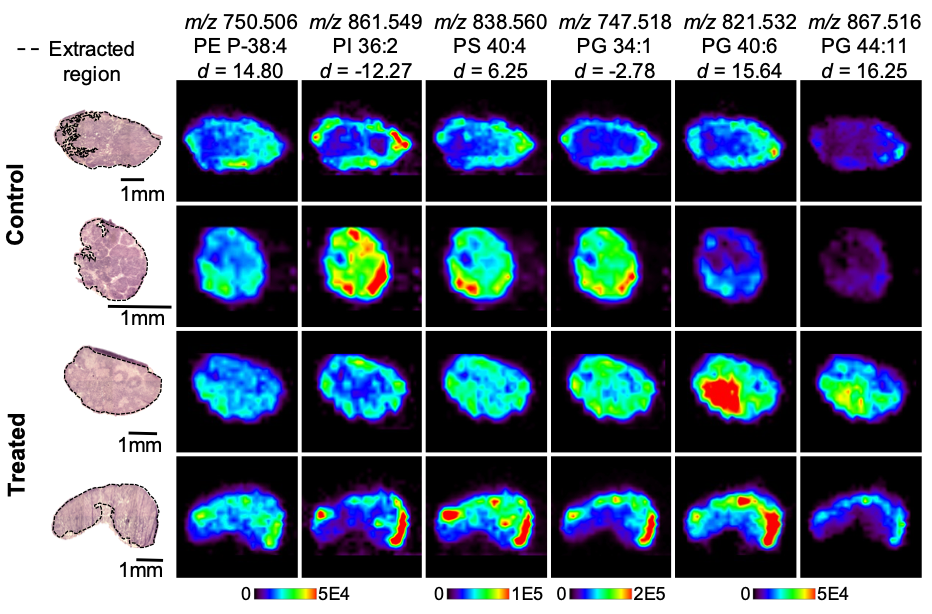


**Fig. S6.** Representative DESI-MS ion images of a subset of PG species that were selected by SAM as significantly different between MC38 treated and control draining lymph node tissues at post-treatment day 15 and also in common with tumor tissues from mice within the same group.


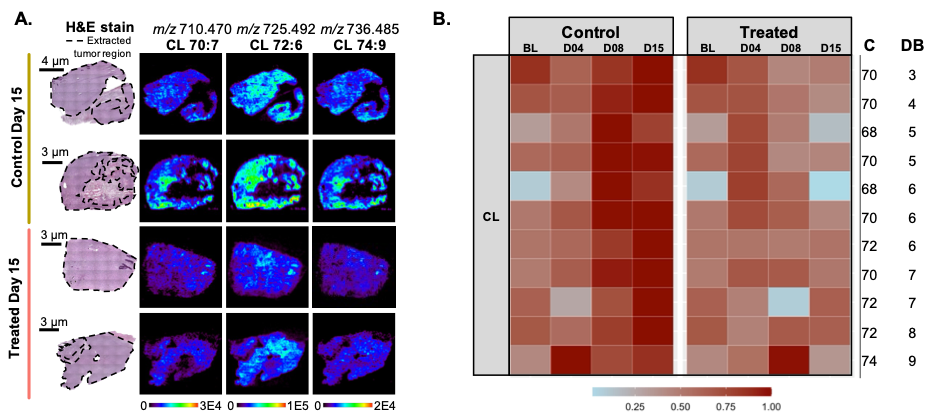


**Fig. S7.** Cardiolipins are decreased in non-responsive anti-PD-1 tumors compared to control tumors in the LL/2 tumor model at post-treatment day 15. **(A)** DESI-MS ion images of a subset of SAM-selected CL species from control (top) and treated (bottom) tumor samples and corresponding H&E-stained tissue sections. **(B)** Heatmap illustrating the change in relative abundances of CL species selected by SAM over time. Baseline (BL) data is repeated twice to facilitate comparison with molecular changes in both treated and control groups over time. Each row represents the normalized intensities of a unique *m/z* value corresponding to a lipid species, averaged for all samples. C is number of carbons and DB is number of double bonds. Red indicates highest normalized intensity while blue indicates low normalized intensity.

**Fig. S8.** Comparison of representative DESI mass spectra obtained from tissue and CD4 and CD8 cell pellets from MC38 tumors at post-treatment day 15. An average of three scans was plotted from regions of highest lipid signal intensity from cell pellet data as well as from highest PUFA lipid signal intensity from tissue. Signal to noise (S/N) was calculated for tentatively identified lipids of interest.

**
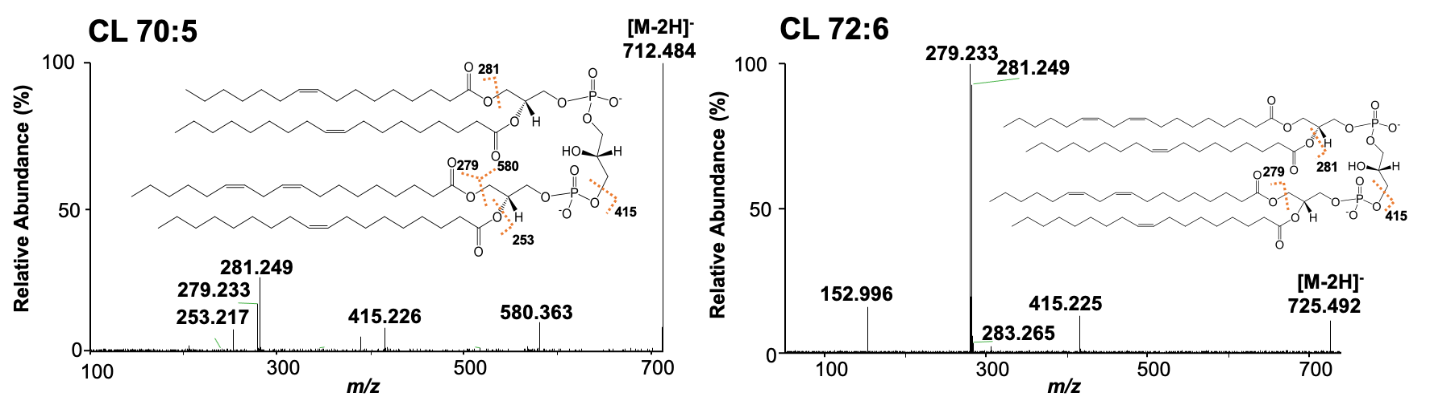
**

**Fig. S9.** Example tandem mass spectra of CL species detected in the LL/2 model which were determined to be significantly different between control tumor and treated tumor both at post-treatment day 15 using SAM.

**Table S1.** Sample numbers for all tissues analyzed by DESI-MS and validated by histopathology for each mouse model and time point.

|  |  | **Baseline** | **Day 4** | | **Day 8** | | **Day 15** | |
| --- | --- | --- | --- | --- | --- | --- | --- | --- |
|  |  |  | Anti-PD-1 | Iso | Anti-PD-1 | Iso | Anti-PD-1 | Iso |
| **Tumor** | **MC38** | 5 | 5 | 4 | 5 | 4 | 3 | 4 |
|  | **MB49** | 4 | 4 | 4 | 3 | 4 | 4 | 4 |
|  | **LL/2** | 5 | 5 | 5 | 5 | 5 | 4 | 5 |
| **Spleen** | **MC38** | 5 | 5 | 5 | 5 | 4 | 5 | 5 |
|  | **MB49** | 4 | 4 | 4 | 4 | 4 | 4 | 4 |
|  | **LL/2** | 5 | 4 | 5 | 5 | 5 | 4 | 5 |
| **Draining lymph node** | **MC38** | 5 | 4 | 5 | 5 | 4 | 5 | 4 |
|  | **MB49** | 4 | 3 | 4 | 4 | 3 | 4 | 4 |
|  | **LL/2** | 5 | 4 | 5 | 4 | 5 | 4 | 5 |

**Table S2.** Tentatively identified SAM features for evaluating lipid differences between treated and control tumors in the MC38 tumor model at post-treatment day 15. The SAM score (d) for molecular features identified as significantly different between treated and control tumors at post-treatment day 4 and day 8 that are also in common with SAM features at post-treatment day 15 are included. SAM features for day 15 that were not also determined as significant for day 4 and day 8 are indicated with “-”.

| **SAM features weighted toward MC38 tumors treated with anti-PD-1 therapy, Day 15** | | | | | | | | | | | | | | | | | | |
| --- | --- | --- | --- | --- | --- | --- | --- | --- | --- | --- | --- | --- | --- | --- | --- | --- | --- | --- |
| **Attribution** | | **Proposed formula** | | **Detected m/z** | | | **Mass error (ppm)** | | **SAM score (d)** | | **Adduct** | | | **Day 4**  **(d)** | | | **Day 8**  **(d)** | |
| **Ceramides** | | | | | | | | | | | | | | | | | | |
| Cer m42:2 | | | C_42_H_81_NO_2_ | | | 666.598 | | 2.85 | | 9.826 | | [M+Cl]^-^ | | | 1.708 | | | 6.490 |
| GluCer d42:2 or GalCer d42:2 | | | C_48_H_91_NO_8_ | | | 844.645 | | 1.42 | | 5.570 | | [M+Cl]^-^ | | | 8.086 | | | 3.394 |
| **Cardiolipins** | | | | | | | | | | | | | | | | | | |
| CL 72:8 | C_81_H_142_O_17_P_2_ | | | 723.479 | | | -0.14 | | 6.250 | | [M-2H]^2-^ | | | - | | | 7.479 | |
| CL 72:7 | C_81_H_144_O_17_P_2_ | | | 724.487 | | | 0.41 | | 5.346 | | [M-2H]^2-^ | | | - | | | 5.770 | |
| **Glycerolipids** | | | | | | | | | | | | | | | | | | |
| DG P-33:4 | C_36_H_62_O_4_ | | | 557.458 | | | 0.18 | | 10.529 | | [M-H]^-^ | | | 4.510 | | | 4.122 | |
| **Phosphatidic acids** | | | | | | | | | | | | | | | | | | |
| PA 34:1 | C_37_H_71_O_8_P | | | 673.481 | | | -0.59 | | 5.727 | | [M-H]^-^ | | | - | | | - | |
| PA 36:4 | C_39_H_69_O_8_P | | | 695.466 | | | 0.43 | | 10.594 | | [M-H]^-^ | | | - | | | - | |
| PA 36:2 | C_39_H_73_O_8_P | | | 699.500 | | | 4.29 | | 5.809 | | [M-H]^-^ | | | 4.641 | | | - | |
| **Phosphatidylethanolamines** | | | | | | | | | | | | | | | | | | |
| PE P-36:4 | C_41_H_74_NO_7_P | | | 722.513 | | | 0.00 | | 10.850 | | [M-H]^-^ | | | -7.535 | | | 14.061 | |
| PE-NMe 36:5 | C_42_H_74_NO_8_P | | | 750.508 | | | 0.13 | | 6.784 | | [M-H]^-^ | | | 2.996 | | | -4.855 | |
| PE 38:4 | C_43_H_78_NO_8_P | | | 766.541 | | | 2.35 | | 6.417 | | [M-H]^-^ | | | -4.443 | | | -3.428 | |
| PE-NMe2 36:1 or PE 38:1 | C_43_H_84_NO_8_P | | | 772.588 | | | 2.33 | | 8.320 | | [M-H]^-^ | | | - | | | -2.493 | |
| PE 40:6 | C_45_H_78_NO_8_P | | | 790.544 | | | 6.07 | | 12.662 | | [M-H]^-^ | | | - | | | - | |
| PE 40:4 or PE-NMe2 38:4 | C_45_H_82_NO_8_P | | | 794.573 | | | 3.15 | | 17.966 | | [M-H]^-^ | | | - | | | 5.330 | |
| **Phosphatidylglycerols** | | | | | | | | | | | | | | | | | | |
| PG 36:5 | C_42_H_73_O_10_P | | | 767.485 | | | -2.48 | | 7.427 | | [M-H]^-^ | | | 9.346 | | | 6.428 | |
| PG 36:4 | C_42_H_75_O_10_P | | | 769.502 | | | -0.65 | | 16.739 | | [M-H]^-^ | | | 8.388 | | | 2.001 | |
| PG 38:7 | C_44_H_73_O_10_P | | | 791.487 | | | 0.13 | | 14.561 | | [M-H]^-^ | | | 9.596 | | | 6.631 | |
| PG 38:6 | C_44_H_75_O_10_P | | | 793.502 | | | -0.63 | | 37.776 | | [M-H]^-^ | | | 16.236 | | | 8.563 | |
| PG 38:5 | C_44_H_77_O_10_P | | | 795.516 | | | -2.77 | | 19.917 | | [M-H]^-^ | | | 13.855 | | | 10.628 | |
| PG 40:8 | C_46_H_75_O_10_P | | | 817.503 | | | 0.61 | | 42.928 | | [M-H]^-^ | | | 13.999 | | | 12.432 | |
| PG 40:7 | C_46_H_77_O_10_P | | | 819.518 | | | -0.24 | | 30.031 | | [M-H]^-^ | | | 14.383 | | | 11.342 | |
| PG 40:6 | C_46_H_79_O_10_P | | | 821.531 | | | -3.41 | | 26.797 | | [M-H]^-^ | | | 15.518 | | | 7.814 | |
| PG 40:5 | C_46_H_81_O_10_P | | | 823.548 | | | -1.82 | | 16.814 | | [M-H]^-^ | | | 12.372 | | | 7.546 | |
| PG 42:10 | C_48_H_75_O_10_P | | | 841.503 | | | 0.59 | | 44.593 | | [M-H]^-^ | | | 11.110 | | | 13.483 | |
| PG 42:9 | C_48_H_77_O_10_P | | | 843.516 | | | -2.61 | | 36.240 | | [M-H]^-^ | | | 15.482 | | | 11.821 | |
| PG 42:8 | C_48_H_79_O_1_0P | | | 845.534 | | | 0.24 | | 39.339 | | [M-H]^-^ | | | 15.204 | | | 10.826 | |
| PG 42:7 | C_48_H_81_O_1_0P | | | 847.549 | | | -0.59 | | 22.945 | | [M-H]^-^ | | | 12.200 | | | 5.473 | |
| PG 44:12 | C_5_0H_75_O_1_0P | | | 865.504 | | | 1.73 | | 39.940 | | [M-H]^-^ | | | 3.812 | | | 10.515 | |
| PG 44:11 | C_5_0H_77_O_1_0P | | | 867.515 | | | -3.69 | | 38.637 | | [M-H]^-^ | | | 10.837 | | | 9.289 | |
| PG 44:10 | C_5_0H_79_O_1_0P | | | 869.533 | | | -0.92 | | 32.675 | | [M-H]^-^ | | | 10.171 | | | 10.709 | |
| **Phosphatidylinositols** | | | | | | | | | | | | | | | | | | |
| PI 34:0 | C_43_H_83_O_13_P | | | 837.547 | | | -3.46 | | 7.723 | | [M-H]^-^ | | | -3.639 | | | 7.389 | |
| PI 40:4 | C_49_H_87_O_13_P | | | 913.581 | | | -0.22 | | 6.505 | | [M-H]^-^ | | | - | | | 7.854 | |
| **Phosphatidylserines** | | | | | | | | | | | | | | | | | | |
| PS 38:4 | C_44_H_78_NO_10_P | | | 810.530 | | | 1.11 | | 24.362 | | [M-H]^-^ | | | -4.856 | | | 6.153 | |
| PS 40:4 | C_46_H_82_NO_10_P | | | 838.562 | | | 1.91 | | 23.268 | | [M-H]^-^ | | | -11.254 | | | 5.193 | |
| **SAM features weighted toward MC38 control tumors, Day 15** | | | | | | | | | | | | | | | | | | |
| **Attribution** | | | **Proposed formula** | | **Detected m/z** | | | **Mass error (ppm)** | | **SAM score (d)** | | **Adduct** | | | **Day 4** | | | **Day 8** |
| **Ceramides** | | | | | | | | | | | | | | | | | | |
| Cer d34:1 | | | C_34_H_67_NO_3_ | | 572.482 | | | 0.35 | | -9.465 | | [M+Cl]^-^ | | | -1.768 | | | - |
| Cer d34:0 | | | C_34_H_69_NO_3_ | | 574.500 | | | 4.18 | | -21.544 | | [M+Cl]^-^ | | | - | | | -4.554 |
| Cer d36:2 | | | C_36_H_69_NO_3_ | | 598.499 | | | 2.17 | | -8.304 | | [M+Cl]^-^ | | | -6.607 | | | - |
| Cer d36:1 | | | C_36_H_71_NO_3_ | | 600.513 | | | 0.83 | | -11.674 | | [M+Cl]^-^ | | | -3.451 | | | -5.277 |
| Cer d38:0 | | | C_38_H_77_NO_3_ | | 630.559 | | | -1.27 | | -6.739 | | [M+Cl]^-^ | | | - | | | - |
| Cer d42:1 | | | C_42_H_83_NO_3_ | | 648.629 | | | -1.54 | | -9.393 | | [M-H]^-^ | | | - | | | - |
| Cer d40:2 | | | C_40_H_77_NO_3_ | | 654.563 | | | 4.89 | | -9.091 | | [M+Cl]^-^ | | | -10.575 | | | 3.415 |
| Cer d40:1 | | | C_40_H_79_NO_3_ | | 656.577 | | | 2.44 | | -10.621 | | [M+Cl]^-^ | | | -2.946 | | | -3.324 |
| Cer d40:0 | | | C_40_H_81_NO_3_ | | 658.594 | | | 4.40 | | -11.852 | | [M+Cl]^-^ | | | - | | | - |
| Cer d42:2 | | | C_42_H_81_NO_3_ | | 682.592 | | | 1.32 | | -12.637 | | [M+Cl]^-^ | | | -8.751 | | | -2.754 |
| Cer d42:1 | | | C_42_H_83_NO_3_ | | 684.607 | | | 0.44 | | -18.296 | | [M+Cl]^-^ | | | - | | | -4.971 |
| Cer d44:1 | | | C_44_H_87_NO_3_ | | 712.641 | | | 4.21 | | -10.877 | | [M+Cl]^-^ | | | 2.613 | | | -1.66 |
| Cer d46:1 | | | C_46_H_91_NO_3_ | | 740.673 | | | 5.00 | | -14.292 | | [M+Cl]^-^ | | | -1.455 | | | -4.283 |
| **Cardiolipins** | | | | | | | | | | | | | | | | | | |
| CL 68:6 | | | C_77_H_138_O_17_P_2_ | | 697.464 | | | 1.15 | | -6.239 | | [M-2H]^2-^ | | | -2.506 | | | 6.205 |
| CL 68:5 | | | C_77_H_140_O_17_P_2_ | | 698.471 | | | -0.86 | | -11.041 | | [M-2H]^2-^ | | | -2.633 | | | 3.714 |
| CL 70:4 | | | C_79_H_146_O_17_P_2_ | | 713.492 | | | -4.20 | | -10.233 | | [M-2H]^2-^ | | | -2.642 | | | - |
| **Glycerolipids** | | | | | | | | | | | | | | | | | | |
| DG O-34:3 | | | C_37_H_68_O_4_ | | 575.503 | | | -4.00 | | -13.831 | | [M-H]^-^ | | | - | | | -2.39 |
| DG 32:1 | | | C_35_H_66_O_5_ | | 601.461 | | | 1.16 | | -16.841 | | [M+Cl]^-^ | | | -5.076 | | | - |
| DG 34:2 | | | C_37_H_68_O_5_ | | 627.477 | | | 1.59 | | -13.758 | | [M+Cl]^-^ | | | -10.227 | | | -5.704 |
| DG 34:1 | | | C_37_H_70_O_5_ | | 629.493 | | | 1.59 | | -15.197 | | [M+Cl]^-^ | | | - | | | 1.433 |
| DG 36:3 | | | C_39_H_70_O_5_ | | 653.494 | | | 3.06 | | -7.644 | | [M+Cl]^-^ | | | -12.717 | | | -5.692 |
| DG 38:5 | | | C_41_H_70_O_5_ | | 677.492 | | | 0.00 | | -6.974 | | [M+Cl]^-^ | | | -8.786 | | | - |
| DG 40:8 | | | C_43_H_68_O_5_ | | 699.476 | | | 0.00 | | -7.05 | | [M+Cl]^-^ | | | -5.846 | | | - |
| TG 36:0 | | | C_39_H_74_O_6_ | | 637.543 | | | 2.67 | | -20.081 | | [M-H]^-^ | | | - | | | - |
| **Fatty acid esters of hydroxy fatty acids** | | | | | | | | | | | | | | | | | | |
| FAHFA O-32:0 | C_32_H_62_O_4_ | | | 509.457 | | | -1.96 | | -12.146 | | [M-H]^-^ | | -1.716 | | | - | | |
| FAHFA O-34:0 | C_34_H_66_O_4_ | | | 537.489 | | | 0.37 | | -11.918 | | [M-H]^-^ | | -3.301 | | | 4.669 | | |
| FAHFA O-36:1 | C_36_H_68_O_4_ | | | 563.505 | | | 0.35 | | -12.718 | | [M-H]^-^ | | 1.97 | | | - | | |
| **Phosphatidylethanolamines** | | | | | | | | | | | | | | | | | | |
| PE 32:1 | C_37_H_72_NO_8_P | | | 688.493 | | | 1.02 | | -6.644 | | [M-H]^-^ | | - | | | - | | |
| PE P-34:2 | C_39_H_74_NO_7_P | | | 698.513 | | | 0.00 | | -12.149 | | [M-H]^-^ | | 6.244 | | | 2.743 | | |
| PE P-34:1 | C_39_H_76_NO_7_P | | | 700.529 | | | 0.43 | | -23.131 | | [M-H]^-^ | | 6.334 | | | 2.325 | | |
| PE P-34:0 | C_39_H_78_NO_7_P | | | 702.544 | | | -0.43 | | -9.635 | | [M-H]^-^ | | 4.809 | | | - | | |
| PE 34:3 | C_39_H_72_NO_8_P | | | 712.486 | | | -8.84 | | -7.897 | | [M-H]^-^ | | - | | | - | | |
| PE 34:2 | C_39_H_74_NO_8_P | | | 714.505 | | | -4.06 | | -15.675 | | [M-H]^-^ | | 5.427 | | | -2.180 | | |
| PE 34:1 | C_39_H_76_NO_8_P | | | 716.523 | | | -0.84 | | -24.258 | | [M-H]^-^ | | 3.116 | | | -4.545 | | |
| PE O-36:4 | C_41_H_76_NO_7_P | | | 724.526 | | | -3.73 | | -14.875 | | [M-H]^-^ | | 2.073 | | | - | | |
| PE O-36:3 | C_41_H_78_NO_7_P | | | 726.544 | | | -0.41 | | -18.813 | | [M-H]^-^ | | 4.347 | | | - | | |
| PE P-36:1 | C_41_H_80_NO_7_P | | | 728.560 | | | 0.00 | | -15.993 | | [M-H]^-^ | | 11.690 | | | - | | |
| PE 36:2 | C_41_H_78_NO_8_P | | | 742.539 | | | -0.27 | | -28.594 | | [M-H]^-^ | | 12.111 | | | -6.424 | | |
| PE 36:1 | C_41_H_80_NO_8_P | | | 744.554 | | | -1.21 | | -21.131 | | [M-H]^-^ | | 4.753 | | | -6.775 | | |
| PE O-38:7 | C_43_H_74_NO_7_P | | | 746.510 | | | -4.02 | | -5.231 | | [M-H]^-^ | | -10.925 | | | 7.049 | | |
| PE P-38:3 | C_43_H_80_NO_7_P | | | 752.559 | | | -1.33 | | -11.686 | | [M-H]^-^ | | - | | | - | | |
| PE 38:5 | C_43_H_76_NO_8_P | | | 764.523 | | | -0.78 | | -12.92 | | [M-H]^-^ | | -10.199 | | | -5.874 | | |
| PE O-38:3 | C_43_H_82_NO_7_P | | | 790.550 | | | -2.91 | | -8.573 | | [M+Cl]^-^ | | - | | | 3.372 | | |
| **Phosphatidylglycerols** | | | | | | | | | | | | | | | | | | |
| LPG 18:1 | C_24_H_47_O_9_P | | | 509.289 | | | 0.98 | | -10.611 | | [M-H]^-^ | | 10.592 | | | - | | |
| PG 32:1 | C_38_H_73_O_10_P | | | 719.487 | | | 0.14 | | -18.624 | | [M-H]^-^ | | -1.580 | | | 1.752 | | |
| PG 32:0 | C_38_H_75_O_10_P | | | 721.503 | | | 0.69 | | -10.029 | | [M-H]^-^ | | -2.148 | | | - | | |
| PG 34:3 | C_40_H_73_O_10_P | | | 743.488 | | | 1.48 | | -5.993 | | [M-H]^-^ | | 5.001 | | | 3.561 | | |
| PG 34:2 | C_40_H_75_O_10_P | | | 745.502 | | | -0.67 | | -19.14 | | [M-H]^-^ | | - | | | 2.337 | | |
| PG 34:1 | C_40_H_77_O_10_P | | | 747.518 | | | -0.27 | | -11.211 | | [M-H]^-^ | | -9.580 | | | 4.724 | | |
| PG 36:2 | C_42_H_79_O_10_P | | | 773.534 | | | 0.26 | | -17.889 | | [M-H]^-^ | | 12.677 | | | 1.579 | | |
| PG 36:1 | C_42_H_81_O_10_P | | | 775.547 | | | -3.22 | | -19.862 | | [M-H]^-^ | | 8.646 | | | 5.315 | | |
| PG 38:3 | C_44_H_81_O_10_P | | | 799.548 | | | -1.88 | | -13.575 | | [M-H]^-^ | | 12.319 | | | 1.276 | | |
| PG 38:2 | C_44_H_83_O_10_P | | | 801.564 | | | -1.37 | | -13.283 | | [M-H]^-^ | | 7.825 | | | - | | |
| **Phosphatidylinositols** | | | | | | | | | | | | | | | | | | |
| LPI 18:0 | C_27_H_53_O_12_P | | | 599.320 | | | 0.33 | | -7.038 | | [M-H]^-^ | | 5.370 | | | - | | |
| PI 34:2 | C_43_H_79_O_13_P | | | 833.519 | | | 0.48 | | -10.815 | | [M-H]^-^ | | 1.783 | | | 3.483 | | |
| PI 36:3 | C_45_H_81_O_13_P | | | 859.533 | | | -1.40 | | -16.623 | | [M-H]^-^ | | -3.223 | | | - | | |
| PI 36:2 | C_45_H_83_O_13_P | | | 861.550 | | | 0.12 | | -20.07 | | [M-H]^-^ | | 16.400 | | | 2.471 | | |
| PI O-38:4 | C_47_H_85_O_12_P | | | 871.570 | | | -0.69 | | -9.776 | | [M-H]^-^ | | -6.556 | | | -4.142 | | |
| **Phosphatidylserines** | | | | | | | | | | | | | | | | | | |
| PS 32:1 | C_38_H_72_NO_10_P | | | 732.483 | | | 1.23 | | -5.969 | | [M-H]^-^ | | -3.860 | | | 2.169 | | |
| PS O-34:3 | C_40_H_74_NO_9_P | | | 742.504 | | | 1.48 | | -5.711 | | [M-H]^-^ | | - | | | -4.369 | | |
| PS 34:2 | C_40_H_74_NO_10_P | | | 758.498 | | | 0.26 | | -6.222 | | [M-H]^-^ | | - | | | 3.451 | | |
| PS 34:1 | C_40_H_76_NO_10_P | | | 760.514 | | | 0.79 | | -11.458 | | [M-H]^-^ | | -2.757 | | | 1.956 | | |
| PS 36:3 | C_42_H_76_NO_10_P | | | 784.513 | | | -0.51 | | -9.513 | | [M-H]^-^ | | 3.833 | | | 2.067 | | |
| PS 38:2 | C_44_H_82_NO_10_P | | | 814.559 | | | -1.72 | | -7.051 | | [M-H]^-^ | | 4.041 | | | 1.310 | | |
| PS 40:2 | C_46_H_86_NO_10_P | | | 842.591 | | | -0.83 | | -9.363 | | [M-H]^-^ | | -4.154 | | | -2.134 | | |
| PS 42:7 | C_48_H_80_NO_10_P | | | 860.537 | | | -8.95 | | -16.017 | | [M-H]^-^ | | 6.338 | | | - | | |
| PS 42:2 | C_48_H_90_NO_10_P | | | 870.623 | | | 0.00 | | -5.166 | | [M-H]^-^ | | - | | | - | | |
| PS 42:1 | C_48_H_92_NO_10_P | | | 872.639 | | | 0.46 | | -5.148 | | [M-H]^-^ | | - | | | - | | |

**Table S3.** Tentatively identified SAM features for evaluating lipid differences between baseline (day 0) and treated tumors (day 15) in the MC38 tumor model. Features that were identified as significant between treated and control tumors at post-treatment day 15 are denoted with “*”.

| **SAM features weighted toward MC38 baseline tumors, Day 0** | | | | | |
| --- | --- | --- | --- | --- | --- |
| **Attribution** | **Proposed formula** | **Detected m/z** | **Mass error (ppm)** | **SAM score (d)** | **Adduct** |
| **Ceramides** | | | | | |
| Cer d34:0 | C_34_H_69_NO_3_ | 574.499* | 3.13 | -9.739 | [M+Cl]^-^ |
| **Cardiolipins** | | | | | |
| CL 68:6 | C_77_H_138_O_17_P_2_ | 697.464* | 1.15 | -6.754 | [M-2H]^2-^ |
| CL 68:5 | C_77_H_140_O_17_P_2_ | 698.471* | -0.86 | -16.795 | [M-2H]^2-^ |
| CL 68:4 | C_77_H_142_O_17_P_2_ | 699.476* | -4.00 | -5.189 | [M-2H]^2-^ |
| CL 70:5 | C_79_H_144_O_17_P_2_ | 712.486* | -0.98 | -17.450 | [M-2H]^2-^ |
| CL 70:4 | C_79_H_146_O_17_P_2_ | 713.493* | -2.80 | -20.438 | [M-2H]^2-^ |
| CL 72:7 | C_81_H_144_O_17_P_2_ | 724.487* | 0.41 | -7.200 | [M-2H]^2-^ |
| CL 68:3 | C_77_H_144_O_17_P_2_ | 700.486 | -1.00 | -5.280 | [M-2H]^2-^ |
| CL 70:7 | C_39_H_70_NO_8_P | 710.471 | 0.00 | -7.197 | [M-2H]^2-^ |
| CL 70:6 | C_79_H_142_O_17_P_2_ | 711.478 | -1.12 | -15.912 | [M-2H]^2-^ |
| **Phosphatidic acids** | | | | | |
| PA 34:1 | C_37_H_71_O_8_P | 673.482* | 0.89 | -15.337 | [M-H]^-^ |
| PA 36:2 | C_39_H_73_O_8_P | 699.499* | 2.86 | -6.185 | [M-H]^-^ |
| PA 34:2 | C_37_H_69_O_8_P | 671.466 | 0.45 | -8.299 | [M-H]^-^ |
| **Phosphatidylethanolamines** | | | | | |
| PE 32:1 | C_37_H_72_NO_8_P | 688.492* | -0.44 | -7.394 | [M-H]^-^ |
| PE P-34:1 | C_39_H_76_NO_7_P | 700.529* | 0.43 | -10.576 | [M-H]^-^ |
| PE 34:2 | C_39_H_74_NO_8_P | 714.505* | -4.06 | -8.967 | [M-H]^-^ |
| PE 34:1 | C_39_H_76_NO_8_P | 716.524* | 0.56 | -19.425 | [M-H]^-^ |
| PE 36:2 | C_41_H_78_NO_8_P | 742.540* | 1.08 | -23.312 | [M-H]^-^ |
| PE 36:1 | C_41_H_80_NO_8_P | 744.554* | -1.21 | -15.239 | [M-H]^-^ |
| PE 38:5 | C_43_H_76_NO_8_P | 764.524* | 0.52 | -12.115 | [M-H]^-^ |
| PE 38:4 | C_43_H_78_NO_8_P | 766.541* | 2.35 | -9.530 | [M-H]^-^ |
| **Phosphatidylglycerols** | | | | | |
| PG 32:1 | C_38_H_73_O_10_P | 719.488* | 1.53 | -17.597 | [M-H]^-^ |
| PG 32:0 | C_38_H_75_O_10_P | 721.502* | -0.69 | -12.588 | [M-H]^-^ |
| PG 34:2 | C_40_H_75_O_10_P | 745.503* | 0.67 | -16.760 | [M-H]^-^ |
| PG 34:1 | C_40_H_77_O_10_P | 747.519* | 1.07 | -21.046 | [M-H]^-^ |
| PG 36:2 | C_42_H_79_O_10_P | 773.534* | 0.26 | -14.835 | [M-H]^-^ |
| **Phosphatidylinositols** | | | | | |
| LPI 18:0 | C_27_H_53_O_12_P | 599.320* | -0.33 | -7.885 | [M-H]^-^ |
| PI 34:2 | C_43_H_79_O_13_P | 833.518* | -0.72 | -14.049 | [M-H]^-^ |
| PI 36:3 | C_45_H_81_O_13_P | 859.534* | -0.23 | -18.274 | [M-H]^-^ |
| PI 36:2 | C_45_H_83_O_13_P | 861.550* | 0.12 | -26.856 | [M-H]^-^ |
| PI 40:4 | C_49_H_87_O_13_P | 913.580* | -1.31 | -10.605 | [M-H]^-^ |
| PI 36:4 | C_45_H_79_O_13_P | 857.519 | 0.47 | -15.893 | [M-H]^-^ |
| PI 38:5 | C_47_H_81_O_13_P | 883.535 | 0.91 | -15.382 | [M-H]^-^ |
| PI 38:4 | C_47_H_83_O_13_P | 885.551 | 1.24 | -20.522 | [M-H]^-^ |
| **Phosphatidylserines** | | | | | |
| PS 32:1 | C_38_H_72_NO_10_P | 732.483* | 1.23 | -19.909 | [M-H]^-^ |
| PS 34:2 | C_40_H_74_NO_10_P | 758.499* | 1.58 | -14.399 | [M-H]^-^ |
| PS 34:1 | C_40_H_76_NO_10_P | 760.514* | 0.79 | -26.586 | [M-H]^-^ |
| PS 36:3 | C_42_H_76_NO_10_P | 784.513* | -0.51 | -11.243 | [M-H]^-^ |
| PS 38:2 | C_44_H_82_NO_10_P | 814.558* | -2.95 | -16.314 | [M-H]^-^ |
| PS 40:4 | C_46_H_82_NO_10_P | 838.561* | 0.72 | -8.399 | [M-H]^-^ |
| PS 40:2 | C_46_H_86_NO_10_P | 842.591* | -0.83 | -14.609 | [M-H]^-^ |
| PS 42:2 | C_48_H_90_NO_10_P | 870.623* | 2.30 | -8.219 | [M-H]^-^ |
| PS 42:1 | C_48_H_92_NO_10_P | 872.638* | -0.69 | -11.400 | [M-H]^-^ |
| PS 36:4 | C_42_H_74_NO_10_P | 782.498 | 0.26 | -7.761 | [M-H]^-^ |
| PS 36:2 | C_42_H_78_NO_10_P | 786.530 | 1.14 | -7.061 | [M-H]^-^ |
| PS 36:1 | C_42_H_80_NO_10_P | 788.545 | 0.38 | -23.028 | [M-H]^-^ |
| PS 38:5 | C_44_H_76_NO_10_P | 808.510 | -4.21 | -7.248 | [M-H]^-^ |
| PS 40:6 | C_46_H_78_NO_10_P | 834.530 | 1.08 | -13.298 | [M-H]^-^ |
| PS 40:1 | C_46_H_88_NO_10_P | 844.605 | -2.72 | -9.979 | [M-H]^-^ |
| **SAM features weighted toward MC38 tumors treated with anti-PD-1 therapy, Day 15** | | | | | |
| **Attribution** | **Proposed formula** | **Detected m/z** | **Mass error (ppm)** | **SAM score (d)** | **Adduct** |
| **Ceramides** | | | | | |
| Cer d34:1 | C_34_H_67_NO_3_ | 572.482* | 0.873 | 7.474 | [M+Cl]^-^ |
| Cer d36:1 | C_36_H_71_NO_3_ | 600.514* | 1.998 | 8.957 | [M+Cl]^-^ |
| Cer d40:2 | C_40_H_77_NO_3_ | 654.561* | 1.833 | 11.876 | [M+Cl]^-^ |
| Cer d40:1 | C_40_H_79_NO_3_ | 656.576* | 0.914 | 13.946 | [M+Cl]^-^ |
| Cer m42:2 | C_42_H_81_NO_2_ | 666.598* | 2.850 | 13.764 | [M+Cl]^-^ |
| Cer d42:2 | C_42_H_81_NO_3_ | 682.592* | 1.319 | 11.420 | [M+Cl]^-^ |
| Cer m42:1 | C_42_H_83_NO_2_ | 668.612 | 0.299 | 10.229 | [M+Cl]^-^ |
| GluCer d34:1 or GalCer d34:1 | C_40_H_77_NO_8_ | 734.535 | 0.953 | 5.675 | [M+Cl]^-^ |
| GluCer d42:2 or GalCer d42:2 | C_48_H_91_NO_8_ | 844.646* | 2.486 | 7.976 | [M+Cl]^-^ |
| **Glycerolipids** | | | | | |
| DG 34:1 | C_37_H_70_O_5_ | 629.494* | 3.177 | 8.647 | [M+Cl]^-^ |
| DG 38:5 | C_41_H_70_O_5_ | 677.491* | -1.476 | 15.140 | [M+Cl]^-^ |
| DG 36:4 | C_39_H_68_O_5_ | 651.477 | 1.381 | 7.924 | [M+Cl]^-^ |
| DG 752 | C_41_H_72_O_5_ | 679.51 | 3.826 | 16.142 | [M+Cl]^-^ |
| **Phosphatidylethanolamines** | | | | | |
| PE P-36:4 | C_41_H_74_NO_7_P | 722.513* | 0.000 | 5.585 | [M-H]^-^ |
| PE-NMe2 36:1 or PE 38:1 | C_43_H_84_NO_8_P | 772.588* | 2.330 | 16.308 | [M-H]^-^ |
| PE 40:6 | C_45_H_78_NO_8_P | 790.544* | 6.072 | 8.869 | [M-H]^-^ |
| PE 40:4 or PE-NMe2 38:4 | C_45_H_82_NO_8_P | 794.573* | 3.146 | 5.967 | [M-H]^-^ |
| PE P-38:4 | C_43_H_78_NO_7_P | 750.543 | -1.732 | 7.695 | [M-H]^-^ |
| **Phosphatidylglycerols** | | | | | |
| PG 36:5 | C_42_H_73_O_10_P | 767.485* | -2.476 | 5.936 | [M-H]^-^ |
| PG 36:4 | C_42_H_75_O_10_P | 769.502* | -0.650 | 10.541 | [M-H]^-^ |
| PG 38:7 | C_44_H_73_O_10_P | 791.487* | 0.126 | 9.965 | [M-H]^-^ |
| PG 38:6 | C_44_H_75_O_10_P | 793.502* | -0.630 | 18.927 | [M-H]^-^ |
| PG 38:5 | C_44_H_77_O_10_P | 795.516* | -2.766 | 12.547 | [M-H]^-^ |
| PG 40:8 | C_46_H_75_O_10_P | 817.503* | 0.612 | 20.567 | [M-H]^-^ |
| PG 40:7 | C_46_H_77_O_10_P | 819.518* | -0.244 | 18.218 | [M-H]^-^ |
| PG 40:6 | C_46_H_79_O_10_P | 821.531* | -3.408 | 18.186 | [M-H]^-^ |
| PG 40:5 | C_46_H_81_O_10_P | 823.548* | -1.821 | 13.830 | [M-H]^-^ |
| PG 42:10 | C_48_H_75_O_10_P | 841.503* | 0.594 | 22.637 | [M-H]^-^ |
| PG 42:9 | C_48_H_77_O_10_P | 843.516* | -2.608 | 18.646 | [M-H]^-^ |
| PG 42:8 | C_48_H_79_O_10_P | 845.534* | 0.237 | 20.832 | [M-H]^-^ |
| PG 42:7 | C_48_H_81_O_10_P | 847.549* | -0.590 | 17.752 | [M-H]^-^ |
| PG 44:12 | C_50_H_75_O_10_P | 865.504* | 1.733 | 18.261 | [M-H]^-^ |
| PG 44:11 | C_50_H_77_O_10_P | 867.515* | -3.689 | 18.575 | [M-H]^-^ |
| PG 44:10 | C_50_H_79_O_10_P | 869.533* | -0.920 | 18.495 | [M-H]^-^ |

**Table S4.** Tentatively identified SAM features for evaluating lipid differences between all treatment-responsive tumors and treatment non-responsive tumors combined regardless of post-treatment time point in the MB49 tumor model. Features that were also identified as significant between treated responder and control tumors in the MC38 responsive model at post-treatment day 15 are denoted with “*”.

| **SAM features weighted toward MB49 Responders** | | | | | |
| --- | --- | --- | --- | --- | --- |
| **Attribution** | **Proposed formula** | **Detected m/z** | **Mass error (ppm)** | **SAM score (d)** | **Adduct** |
| **Ceramides** | | | | | |
| Cer d34:1 | C_34_H_67_NO_3_ | 572.482* | 0.87 | 10.558 | [M+Cl]^-^ |
| Cer m42:2 | C_42_H_81_NO_2_ | 666.598* | 2.85 | 5.525 | [M+Cl]^-^ |
| **Cardiolipins** | | | | | |
| CL 72:8 | C_81_H_142_O_17_P_2_ | 723.48* | 1.24 | 17.103 | [M-2H]^2-^ |
| **Glycerolipids** | | | | | |
| DG 34:2 | C_37_H_68_O_5_ | 627.476* | 0.00 | 9.008 | [M+Cl]^-^ |
| DG 36:3 | C_39_H_70_O_5_ | 653.494* | 3.06 | 15.297 | [M+Cl]^-^ |
| DG 36:4 | C_39_H_68_O_5_ | 651.476 | -0.15 | 19.498 | [M+Cl]^-^ |
| DG 38:4 | C_41_H_72_O_5_ | 679.51 | 3.83 | 28.205 | [M+Cl]^-^ |
| DG 38:5 | C_41_H_70_O_5_ | 677.492* | 0.00 | 12.975 | [M+Cl]^-^ |
| DG P-33:4 | C_36_H_62_O_4_ | 557.458* | 0.90 | 10.341 | [M-H]^-^ |
| **Phosphatidyethanolamines** | | | | | |
| PE 38:4 | C_43_H_78_NO_8_P | 766.539* | -0.26 | 25.426 | [M-H]^-^ |
| PE 40:4 or PE-NMe2 38:4 | C_45_H_82_NO_8_P | 794.571* | 0.63 | 15.130 | [M-H]^-^ |
| PE 40:6 | C_45_H_78_NO_8_P | 790.54* | 1.01 | 20.211 | [M-H]^-^ |
| PE P-36:4 | C_41_H_74_NO_7_P | 722.514* | 1.38 | 32.777 | [M-H]^-^ |
| PE P-38:4 | C_43_H_78_NO_7_P | 750.543 | -1.73 | 28.889 | [M-H]^-^ |
| PE-NMe 36:5 | C_42_H_74_NO_8_P | 750.507* | -1.20 | 11.061 | [M-H]^-^ |
| **Phosphatidylglycerols** | | | | | |
| PG 36:4 | C_42_H_75_O_10_P | 769.503* | 0.65 | 26.540 | [M-H]^-^ |
| PG 38:5 | C_44_H_77_O_10_P | 795.518* | -0.25 | 5.594 | [M-H]^-^ |
| PG 38:6 | C_44_H_75_O_10_P | 793.501* | -1.89 | 31.168 | [M-H]^-^ |
| PG 38:7 | C_44_H_73_O_10_P | 791.489* | 2.65 | 5.325 | [M-H]^-^ |
| PG 40:5 | C_46_H_81_O_10_P | 823.550* | 0.61 | 5.877 | [M-H]^-^ |
| PG 40:6 | C_46_H_79_O_10_P | 821.531* | -3.41 | 27.014 | [M-H]^-^ |
| PG 40:7 | C_46_H_77_O_10_P | 819.519* | 0.98 | 15.083 | [M-H]^-^ |
| PG 40:8 | C_46_H_75_O_10_P | 817.504* | 1.83 | 36.995 | [M-H]^-^ |
| PG 42:10 | C_48_H_75_O_10_P | 841.504* | 1.78 | 31.955 | [M-H]^-^ |
| PG 42:8 | C_48_H_79_O_10_P | 845.534* | 0.24 | 24.123 | [M-H]^-^ |
| PG 42:9 | C_48_H_77_O_10_P | 843.516* | -2.61 | 9.844 | [M-H]^-^ |
| PG 44:10 | C_50_H_79_O_10_P | 869.533* | -0.92 | 17.486 | [M-H]^-^ |
| PG 44:12 | C_50_H_75_O_10_P | 865.503* | 0.58 | 32.505 | [M-H]^-^ |
| **Phosphatidylinositols** | | | | | |
| LPI 18:0 | C_27_H_53_O_12_P | 599.322* | 3.00 | 25.249 | [M-H]^-^ |
| PI 34:0 | C_43_H_83_O_13_P | 837.545* | -5.85 | 10.638 | [M-H]^-^ |
| PI 36:4 | C_45_H_79_O_13_P | 857.520 | 1.63 | 15.560 | [M-H]^-^ |
| PI 38:4 | C_47_H_83_O_13_P | 885.551 | 1.24 | 17.586 | [M-H]^-^ |
| PI 40:4 | C_49_H_87_O_13_P | 913.582* | 0.88 | 12.534 | [M-H]^-^ |
| **Phosphatidylserines** | | | | | |
| PS 36:2 | C_42_H_78_NO_10_P | 786.530 | 1.14 | 14.985 | [M-H]^-^ |
| PS 36:4 | C_42_H_74_NO_10_P | 782.499 | 1.53 | 8.656 | [M-H]^-^ |
| PS 38:4 | C_44_H_78_NO_10_P | 810.530* | 1.11 | 25.406 | [M-H]^-^ |
| PS 38:5 | C_44_H_76_NO_10_P | 808.512 | -1.73 | 8.802 | [M-H]^-^ |
| PS 40:4 | C_46_H_82_NO_10_P | 838.562* | 1.91 | 32.607 | [M-H]^-^ |
| PS 40:6 | C_46_H_78_NO_10_P | 834.530 | 1.08 | 25.223 | [M-H]^-^ |
| **SAM features weighted toward MB49 Non-responders** | | | | | |
| **Attribution** | **Proposed formula** | **Detected m/z** | **Mass error (ppm)** | **SAM score (d)** | **Adduct** |
| **Ceramides** | | | | | |
| Cer d40:0 | C_40_H_81_NO_3_ | 658.593* | 2.88 | -12.308 | [M+Cl]^-^ |
| Cer d42:1 | C_42_H_83_NO_3_ | 684.607* | 0.44 | -20.686 | [M+Cl]^-^ |
| Cer d44:1 | C_44_H_87_NO_5_ | 708.649 | -3.10 | -6.699 | [M-H]^-^ |
| Cer d34:0 | C_34_H_69_NO_3_ | 574.497* | -0.35 | -8.191 | [M+Cl]^-^ |
| Cer d42:1 | C_42_H_83_NO_3_ | 648.629* | -1.54 | -15.016 | [M-H]^-^ |
| Cer m42:1 | C_42_H_83_NO_2_ | 668.613 | 1.79 | -5.176 | [M+Cl]^-^ |
| **Cardiolipins** | | | | | |
| CL 68:5 | C_77_H_140_O_17_P_2_ | 698.471* | -0.86 | -22.153 | [M-2H]^2-^ |
| CL 68:6 | C_77_H_138_O_17_P_2_ | 697.464* | 1.15 | -8.248 | [M-2H]^2-^ |
| CL 70:4 | C_79_H_146_O_17_P_2_ | 713.493* | -2.80 | -23.662 | [M-2H]^2-^ |
| CL 70:6 | C_79_H_142_O_17_P_2_ | 711.477 | -2.53 | -28.852 | [M-2H]^2-^ |
| CL 70:7 | C_79_H_140_O_17_P_2_ | 710.470 | -1.41 | -16.711 | [M-2H]^2-^ |
| CL 72:5 | C_81_H_148_O_17_P_2_ | 726.500 | -3.17 | -22.914 | [M-2H]^2-^ |
| CL 72:6 | C_81_H_146_O_17_P_2_ | 725.493 | -2.07 | -17.669 | [M-2H]^2-^ |
| CL 72:7 | C_40_H_72_NO_8_P | 724.485* | -9.94 | -11.755 | [M-2H]^2-^ |
| **Glycerolipids** | | | | | |
| DG 32:1 | C_35_H_66_O_5_ | 601.460* | -0.50 | -7.571 | [M+Cl]^-^ |
| DG 34:1 | C_37_H_70_O_5_ | 629.493* | 1.59 | -8.698 | [M+Cl]^-^ |
| DG 38:2 | C_41_H_76_O_5_ | 683.540 | 1.90 | -15.467 | [M+Cl]^-^ |
| DG 42:8 | C_45_H_72_O_5_ | 727.507 | -0.55 | -10.012 | [M+Cl]^-^ |
| TG 36:0 | C_39_H_74_O_6_ | 637.54* | -2.04 | -18.826 | [M-H]^-^ |
| **Fatty acid esters of hydroxy fatty acids** | | | | | |
| FAHFA O-36:1 | C_36_H_68_O_4_ | 563.503* | -2.48 | -12.284 | [M-H]^-^ |
| **Phosphatic acids** | | | | | |
| PA 34:1 | C_37_H_71_O_8_P | 673.48* | -2.08 | -7.719 | [M-H]^-^ |
| **Phosphatidylethanolamines** | | | | | |
| PE 34:1 | C_39_H_76_NO_8_P | 716.524* | 0.56 | -19.761 | [M-H]^-^ |
| PE 36:1 | C_41_H_80_NO_8_P | 744.552* | -3.89 | -15.829 | [M-H]^-^ |
| PE 36:2 | C_41_H_78_NO_8_P | 742.538* | -1.62 | -16.372 | [M-H]^-^ |
| PE 38:2 | C_43_H_82_NO_8_P | 770.569 | -1.95 | -12.803 | [M-H]^-^ |
| PE O-36:3 | C_41_H_78_NO_7_P | 726.541* | -4.54 | -22.441 | [M-H]^-^ |
| PE O-36:4 | C_41_H_76_NO_7_P | 724.526* | -3.73 | -8.525 | [M-H]^-^ |
| PE P-34:0 | C_39_H_78_NO_7_P | 702.542* | -3.27 | -18.169 | [M-H]^-^ |
| PE P-34:1 | C_39_H_76_NO_7_P | 700.528* | -1.00 | -23.392 | [M-H]^-^ |
| PE P-36:1 | C_41_H_80_NO_7_P | 728.558* | -2.75 | -24.196 | [M-H]^-^ |
| **Phosphatidylglycerols** | | | | | |
| LPG 18:1 | C_24_H_47_O_9_P | 509.289* | 0.98 | -21.345 | [M-H]^-^ |
| PG 32:0 | C_38_H_75_O_10_P | 721.502* | -0.69 | -9.512 | [M-H]^-^ |
| PG 32:1 | C_38_H_73_O_10_P | 719.486* | -1.25 | -21.608 | [M-H]^-^ |
| PG 34:1 | C_40_H_77_O_10_P | 747.517* | -1.61 | -22.612 | [M-H]^-^ |
| PG 34:2 | C_40_H_75_O_10_P | 745.501* | -2.01 | -29.829 | [M-H]^-^ |
| PG 36:1 | C_42_H_81_O_10_P | 775.546* | -4.51 | -15.095 | [M-H]^-^ |
| PG 36:2 | C_42_H_79_O_10_P | 773.532* | -2.33 | -32.831 | [M-H]^-^ |
| PG 38:2 | C_44_H_83_O_10_P | 801.563* | -2.62 | -30.947 | [M-H]^-^ |
| PG 38:3 | C_44_H_81_O_10_P | 799.547* | -3.13 | -15.776 | [M-H]^-^ |
| **Phosphatidylinositols** | | | | | |
| PI 34:2 | C_43_H_79_O_13_P | 833.518* | -0.72 | -5.730 | [M-H]^-^ |
| PI 36:2 | C_45_H_83_O_13_P | 861.548* | -2.21 | -23.399 | [M-H]^-^ |
| PI 36:3 | C_45_H_81_O_13_P | 859.531* | -3.72 | -10.048 | [M-H]^-^ |
| **Phosphatidylserines** | | | | | |
| PS 34:1 | C_40_H_76_NO_10_P | 760.512* | -1.84 | -23.255 | [M-H]^-^ |
| PS 36:3 | C_42_H_76_NO_10_P | 784.513* | -0.51 | -5.790 | [M-H]^-^ |
| PS 38:2 | C_44_H_82_NO_10_P | 814.559* | -1.72 | -17.939 | [M-H]^-^ |
| PS 40:2 | C_46_H_86_NO_10_P | 842.59* | -2.02 | -21.446 | [M-H]^-^ |

**Movie S1 (separate file).** DESI-MS ion image of PG 40:8 (*m/z* 817.501) overlaid with merged IF image (CD3, green; CD45, red; DAPI, blue). Scale bar for IF image is 1.0 mm.
